# Voltage-Gated Ion Channels in Neuropathic Pain Signaling

**DOI:** 10.3390/life15060888

**Published:** 2025-05-30

**Authors:** Ricardo Felix, Alejandra Corzo-Lopez, Alejandro Sandoval

**Affiliations:** 1 Department of Cell Biology, Centre for Research and Advanced Studies (Cinvestav), Mexico City 07360, Mexico; 2 School of Medicine FES Iztacala, National Autonomous University of Mexico (UNAM), Tlalnepantla 54090, Mexico

**Keywords:** voltage-gated ion channels, neuropathic pain, calcium channels, Ca_V_ channels, potassium channels, K_V_ channels, sodium channels, Na_V_ channels, PROTACs

## Abstract

Neuropathic pain is a chronic and debilitating disorder of the somatosensory system that affects a significant proportion of the population and is characterized by abnormal responses such as hyperalgesia and allodynia. Voltage-gated ion channels, including sodium (Na_V_), calcium (Ca_V_), and potassium (K_V_) channels, play a pivotal role in modulating neuronal excitability and pain signal transmission following nerve injury. This review intends to provide a comprehensive analysis of the molecular and cellular mechanisms by which dysregulation in the expression, localization, and function of specific Na_V_ channel subtypes (mainly Na_V_1.7 and Na_V_1.8) and their auxiliary subunits contributes to aberrant neuronal activation, the generation of ectopic discharges, and sensitization in neuropathic pain. Likewise, special emphasis is placed on the crucial role of Ca_V_ channels, particularly Ca_V_2.2 and the auxiliary subunit Ca_V_α_2_δ, whose overexpression increases calcium influx, neurotransmitter release, and neuronal hyperexcitability, thus maintaining persistent pain states. Furthermore, K_V_ channels (particularly K_V_7 channels) function as brakes on neuronal excitability, and their dysregulation facilitates the development and maintenance of neuropathic pain. Therefore, targeting specific K_V_ channel subtypes to restore their function is also a promising therapeutic strategy for alleviating neuropathic pain symptoms. On the other hand, recent advances in the development of small molecules as selective modulators or inhibitors targeting voltage-gated ion channels are also discussed. These agents have improved efficacy and safety profiles in preclinical and clinical studies by attenuating pathophysiological channel activity and restoring neuronal function. This review seeks to contribute to guiding future research and drug development toward more effective mechanism-based treatments by discussing the molecular mechanisms underlying neuropathic pain and highlighting translational therapeutic opportunities.

## 1. Introduction

**Figure 1:**
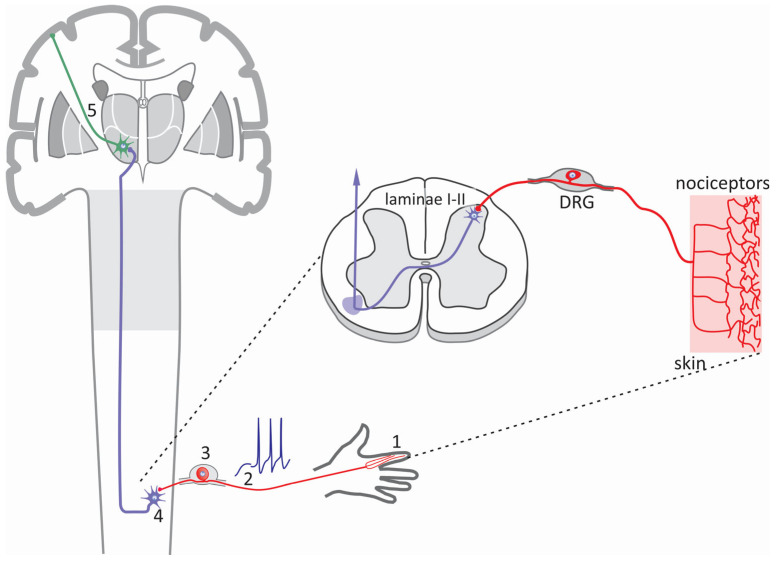
The sensory pathway. The somatosensory system comprises an intricate network of sensory receptors distributed throughout the skin, muscles, joints, and internal organs. These receptors include nociceptors activated in response to noxious stimuli and generate pain signals. The information generated in the periphery (1) is transmitted as action potentials (2), primarily via primary afferent fibers of the Aδ and C type that have a peripheral axon innervating the distal regions, to the DRG (3), where the soma of the sensory neurons are located. The pain signals then travel to the second-order neurons in the laminae I-II of the spinal cord (4). Finally, these signals are transmitted to third-order neurons in the thalamus (5) and then to the primary somatosensory cortex to be integrated.

The somatosensory system enables us to perceive and interact with our environment and body through highly specialized peripheral sensory neurons in the skin, muscles, joints, and internal organs that detect touch, pressure, pain, and temperature. These neurons detect environmental stimuli, convert them into action potentials (APs), and transmit them to the brain via the spinal cord (SC). These same neurons transmit pain signals through the peripheral nerves to the SC, where second-order neurons transfer them to the thalamus. The thalamus receives these signals and projects them to the primary somatosensory cortex, where the information is integrated [1] (Figure 1).

Neuropathic pain is a disorder of the somatosensory system that affects ~10% of the general population. It is more frequent in women and in individuals over 50 years of age and most frequently affects the lower back, upper and lower limbs, and the neck [2,3]. It is characterized by abnormal responses to stimuli, including hyperalgesia, an increased painful response to painful stimuli, and allodynia, the presence of pain associated with innocuous stimuli. Different conditions may cause neuropathic pain, with diabetes mellitus being one of the most significant [3]. However, neuropathic pain may also occur as a result of herpes virus infections, acquired immunodeficiency syndrome, or have a traumatic origin. Likewise, autoimmune disorders such as Guillain–Barré syndrome and multiple sclerosis, as well as some oncological treatments, may also cause peripheral neuropathy [1,3].

It is well-known that the changes responsible for neuropathic pain mechanisms lie in altered gene transcription or protein functional expression/localization in sensory neurons. Interestingly, after damage to peripheral sensory fibers, alterations in the different subunits that compose voltage-gated ion channels may contribute to the changes in pain transmission observed in allodynia and hyperalgesia, as we shall discuss next [1,4].

## 2. Voltage-Gated Sodium (Na_V_) Channels and Neuropathic Pain

### 2.1. Structure and Function of Na_V_ Channels

Na_V_ channels play a relevant role in the development and maintenance of neuropathic pain. These proteins are essential components of the excitability machinery in excitable cells, including neurons, and alterations in their expression or function after nerve injury may cause greater pain sensitivity. When the receptor potential is sufficient to reach the activation threshold of Na_V_ channels, it will trigger the generation and propagation of regenerative action potentials (APs) in nociceptive neurons and the transmission of pain signals to the SC [3,4].

**Figure 2:**
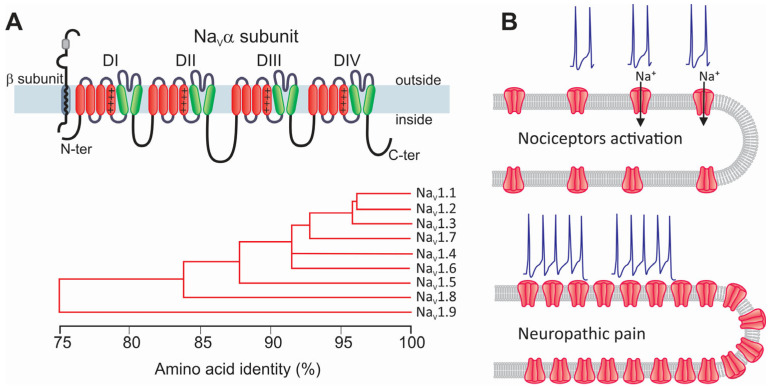
Structure and classification of Na_V_ channels and their participation in neuropathic pain. (**A**) The α subunit forms the ion-conducting region of Na_V_ channels. This protein comprises four repeated homologous domains (DI–DIV), formed by six transmembrane segments connected by intracellular loops. Segment S4 (+) acts as the channel voltage sensor. Although the Na_V_α subunit alone can form a functional channel, it is generally associated with auxiliary subunits β (blue; Na_V_β1–Na_V_β4) that regulate its biophysical properties, its trafficking to the membrane, and its interaction with proteins extrinsic to the channel (upper panel). Nine α subunits have been identified (Na_V_1.1α–Na_V_1.9α) that share a similar membrane topology, each encoded by a different gene with different properties. The phylogenetic tree illustrates the amino acid sequence similarity of the mammal Na_V_α subunits encoding the nine identified Na_V_ channels (lower panel). (**B**) In neuropathic pain, significant alterations occur in both the functional expression and the activity of Na_V_ channels. These changes affect neuronal excitability, which translates as increased sensitivity to pain and produces hyperalgesia and allodynia.

Na_V_ channels are multimeric complexes formed by a main subunit (Na_V_α) that forms the ion-conducting pore (~250 kD), together with one or two auxiliary Na_V_β subunits (30–40 kD) (Figure 2A). Na_V_α subunits are encoded by ten genes (SCN) that give rise to nine different proteins named Na_V_1.1–Na_V_1.9 and a novel subfamily known as Na_V_x [5,6,7] (Figure 2A). These proteins comprise twenty-four transmembrane segments organized into four homologous repeat domains, each containing six transmembrane segments. Likewise, the fourth of these segments, S4, is positively charged and considered as the region that serves as a sensor for transmembrane voltage changes. The loop that connects the S5 and S6 transmembrane segments forms the channel’s pore [6,7] (Figure 2A).

On the other hand, the auxiliary subunits of Na_V_ channels (Na_V_β1 to Na_V_β4) are glycoproteins containing one single transmembrane segment with a small intracellular domain and an extracellular region similar to that of cell adhesion molecules [8,9]. Na_V_α subunits alone are sufficient to reconstitute the sodium channel function. In contrast, Na_V_β subunits modulate the voltage dependence and kinetics of the currents that flow through the pore-forming subunit [6,8,9,10].

### 2.2. The Role of Na_V_ Channels in the Pathogenesis of Neuropathic Pain

Over the past few years, some mutations in Na_V_ channel proteins and changes in their functional expression and post-translational processing have been associated with neuropathic pain [11,12,13,14,15]. Injuries or conditions affecting the peripheral nerves produce axonopathy and demyelination. This is because neuropathy alters the patterns of AP firing due to the remodeling of the neuron’s intrinsic electrical properties [16,17] (Figure 2B). Animal models of neuropathic pain have shown that after peripheral nerve injury, Na_V_ channels are relocated, which is accompanied by changes in neuronal excitability that produce the ectopic activation of APs, ultimately leading to allodynia and hyperalgesia. After nerve injury, the percentage of neurons that can generate APs persistently and significantly increases. In parallel, this change causes subthreshold membrane potential oscillations and the emergence of pacemaker activity [17,18,19,20]. Augmented neuronal excitability and consequent increased discharge are the primary signals of neuropathic pain and trigger central sensitization [1,21].

Na_V_ channels are concentrated in the proximal segment and nodes of Ranvier of healthy sensory neurons; however, after injury, it has been noted that Na_V_1.7 and Na_V_1.8 channels tend to concentrate at the axonal ends of neuromas generated at the site of injury, altering the excitability of injured cells [22,23]. This occurs due to a local buildup of vesicles transporting Na_V_ channels. Some of these channels reach the cell membrane at the injury site, increasing their number and altering local electrogenic properties (Figure 2B).

However, in addition to changes in the localization of Na_V_ channels, gene transcription is also regulated in dorsal root ganglion (DRG) sensory neurons during neuropathic pain [24,25]. In this regard, it is known that Na_V_1.1-, Na_V_1.2-, and Na_V_1.3-type channels are expressed in DRG neurons only in the embryonic stage, while adult cells predominantly express Na_V_1.7, Na_V_1.8, and Na_V_1.9, and to a lesser extent, Na_V_1.1 and Na_V_1.6 [26]. During neuropathic pain, there is a change in the expression profile of Na_V_ channels that significantly impacts sensory neurons’ excitability by producing a hyperpolarization of the resting membrane potential [27]. This, in turn, allows Na_V_ channels to transition from the inactivated refractory state to the closed state, increasing the fraction of channels available to be activated.

Sensitive to tetrodotoxin (TTX), Na_V_1.1 channels are crucial in the central nervous system (CNS) because they are key to generating and propagating APs. Likewise, given that their activity contributes to determining neurons’ activation threshold, the proper functioning of these proteins prevents the neuronal hyperexcitability that accompanies diverse CNS disorders, including neuropathic pain. Indeed, pharmacological inhibition of these channels has been shown to reduce mechanical pain in a peripheral nerve injury model [28]. In addition, from a molecular perspective, experimental evidence suggests an increased expression of Na_V_1.1 channels in DRG neurons shortly after nerve injury, both at the RNA and protein level, pointing to their possible role in the abnormal neuronal activity associated with neuropathic pain [29].

As mentioned earlier, Na_V_1.3 channels mediate a rapidly activating and inactivating current sensitive to TTX associated with neuropathic pain in axotomized DRG neurons [30]. These channels are expressed at low levels in adult primary sensory afferents, but are rapidly upregulated in DRG neurons after peripheral or spinal nerve injury or ligation [31,32,33,34].

Interestingly, it has been reported that the expression pattern of Na_V_ channels undergoes an important change in the neuropathic pain model resulting from nerve injury. Indeed, this expression pattern changes from an increased expression of tetrodotoxin-resistant channels (TTX-R; Na_V_1.8 and Na_V_1.9) to an increased expression of toxin-sensitive channels (TTX-S; Na_V_1.3) in injured neurons [26,31,33,35]. In this context, it has been proposed that the augmentation in excitability observed in injured neurons is the result, at least in part, of this increase in the expression of Na_V_1.3 channels [36,37].

This idea is grounded in the fact that the biophysical properties of these channels are suitable for promoting spontaneous ectopic discharge, which, as mentioned above, is a hallmark of injured nerves. Although it is generally accepted that Na_V_1.3 channels may participate in initiating and maintaining neuropathic pain [36,37], unexpectedly, studies in Na_V_1.3 knockout animals have reported that such animals can develop neuropathic pain after peripheral nerve injury [38,39]. The origin of this difference is unknown, though it may lie in the changes observed in the expression patterns of other Na_V_ channels, as we shall discuss next.

Na_V_1.7 channels are primarily expressed in nociceptive neurons and are essential in generating APs in response to noxious stimuli [23]. Regarding its role in the development of neuropathic pain, it has been speculated that nerve injury may lead to the upregulation of Na_V_1.7 channels, which would contribute to an increase in neuronal excitability and ectopic discharges, which are characteristic features of neuropathic pain [40]. However, the exact mechanisms by which Na_V_1.7 channels influence neuropathic pain remain poorly understood, and some studies suggest that compensatory mechanisms might mask their role in specific cellular contexts.

It has been hypothesized that nerve growth factor (NGF) may play an important role in the increased expression of Na_V_1.7 channels observed after nerve injury. Elevated levels of NGF lead to an increased expression of these channels through signaling pathways involving various transcription factors. These molecular events may promote the expression of Na_V_1.7 channels and eventually contribute to the general sensitization process and the maintenance of neuropathic pain [23,40].

**Figure 3:**
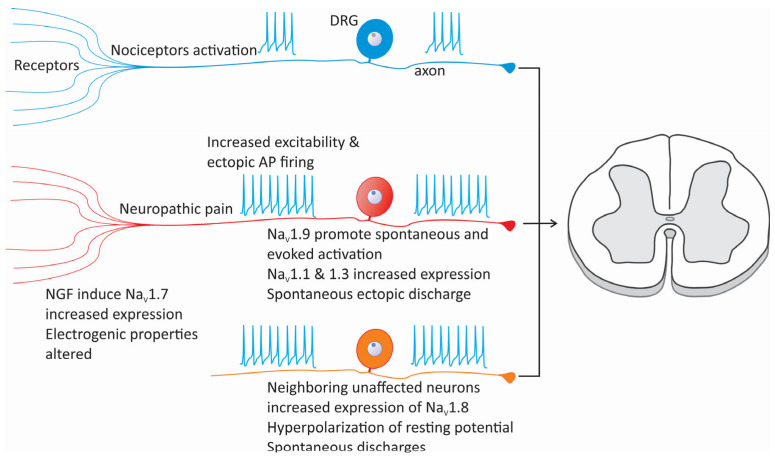
Changes in Na_V_ channel expression and cellular excitability in neuropathic pain. Alterations in the expression of various subtypes of sodium channels, such as Na_V_1.1, Na_V_1.3, Na_V_1.7, Na_V_1.8, and Na_V_1.9, can increase cellular excitability, reducing the activation threshold of nociceptors. Similarly, after nerve injury, neurons adjacent to the damaged area may experience changes in the expression of Na_V_ channels, particularly Na_V_1.3 and Na_V_1.8, which causes the development of ectopic foci of neuronal activity. On the other hand, the expression of Na_V_1.8 channels in neurons neighboring an injured nerve can also be compromised, contributing to the maintenance of neuropathic pain.

Like other voltage-gated sodium channels, Na_V_1.8 plays an essential role in developing and maintaining neuropathic pain through its effects on neuronal excitability and the generation of abnormal electrical activity in sensory neurons [41]. After nerve injury, the expression of these channels is often significantly affected. Initially, Na_V_1.8 expression may be downregulated in injured neurons, though neighboring uninjured neurons often show increased expression [23]. In addition, the biophysical properties of Na_V_1.8 channels may also be affected after nerve injury. Changes in the properties of Na_V_ channels can shift the activation curve toward hyperpolarized values, lowering the threshold for firing APs. This makes neurons more likely to activate repeatedly [42] (Figure 3). These changes may significantly contribute to the spontaneous discharges seen in hyperalgesia associated with neuropathic pain. Notably, changes in the expression of Na_V_1.7 and Na_V_1.8 channels may exacerbate neuronal excitability by converging via complex cell mechanisms to determine the persistence of neuropathic pain [43].

Following nerve injury, cross-talk can occur between intact and injured neurons, where changes in Na_V_1.8 channel expression can influence the activity of nearby neurons expressing Na_V_1.7 channels (Figure 3). As mentioned above, the initially low expression of Na_V_1.8 channels in injured cells is increased in neighboring uninjured neurons, which ultimately results in increased Na_V_1.8 expression, generating ectopic activity. On the other hand, the upregulation of Na_V_1.7 channel expression in these cells increases the neuronal excitability and ectopic AP firing caused by changes in Na_V_1.8 expression [43]. Together, these events amplify pain signaling by causing a state of persistent neuronal hyperexcitability.

Finally, it has been proposed that Na_V_ channel mRNA may be transported peripherally from DRGs to the sciatic nerve and translated locally. In particular, the local upregulation of Na_V_1.8 channel mRNA has been observed after nerve injury, which may contribute, at least in part, to the increase in channel protein levels observed at the peripheral level and in neuronal excitability. This, in turn, may play a relevant role in the aberrant nociception that characterizes neuropathic pain [44].

Na_V_1.9 channels are expressed primarily in nociceptive neurons, where they play a relevant role in pain signaling, especially in neuropathic pain conditions [24,27]. In addition to those reviewed in the preceding sections, these channels generate APs and amplify pain signals, especially after nerve injury. While Na_V_1.9 channels mediate TTX-R sodium currents that are crucial for maintaining neuronal excitability under physiological conditions [41,45], under pathological conditions, sodium currents through these channels can become dysregulated and contribute to chronic pain states. Numerous studies suggest that Na_V_1.9 channels favor spontaneous and evoked activation in DRG neurons, which, in abnormal conditions, leads to exacerbated pain perception [24,27].

### 2.3. The Role of the Na_V_ Channel Auxiliary Subunits in Neuropathic Pain

Furthermore, as already mentioned, Na_V_β auxiliary subunits regulate the kinetic properties and voltage dependence of the ion-conducting subunits of Na_V_ channels [9,46,47]. Therefore, alterations in the functional expression of these proteins may influence the development of neuropathic pain due to their central role in the excitability of sensory neurons. On the other hand, it has also been observed that the expression of the auxiliary subunits Na_V_β_2_ and Na_V_β_3_ can be augmented after peripheral nerve injury, both in injured sensory neurons and neighboring uninjured nerve cells. This could be associated with neuronal hyperexcitability and the development of ectopic activity [48].

Previous work on Na_V_β_2_ subunit expression has revealed that this protein is upregulated following peripheral nerve injury, which may affect neuronal excitability [48]. This idea is supported by results obtained in Na_V_β_2_ null mice, showing decreased Na_V_ TTX-S channel expression in DRG neurons [10,49]. Remarkably, mechanical allodynia associated with peripheral nerve injury was attenuated in the knockout animals, consistent with the role of this protein in neuropathic pain [10,49].

The auxiliary subunit Na_V_β_3_ may also play a role in neuropathic pain. As discussed above, there is evidence that peripheral nerve injury induces an increase in currents passing through TTX-S Na_V_ channels related to an increase in the expression of Na_V_1.3 in DRG neurons. This occurs in parallel with increased Na_V_β_3_ mRNA and protein levels [50,51]. It is worth noting that the co-expression of the Na_V_β_3_ subunit in heterologous expression systems produces changes in the activation and inactivation of Na_V_1.3 channels, faster recovery from inactivation, and slower kinetics of the current [10,52]. Likewise, the overexpression of *Scn3b* mRNA has been observed in multiple pain models, specifically depending on the type of fiber and the model used. In a chronic injury model, it was increased in C fibers, while in a diabetic neuropathy model, it was increased in medium Aδ fibers and the lumbar SC [50,51].

Therefore, it has been speculated that the overexpression of Na_V_1.3 channels and Na_V_β_3_ subunits represents an attempt by sensory neurons to compensate for the decrease in the expression of Na_V_1.8 and Na_V_1.9 channels induced by nerve injury [25,32]. This causes alterations in the properties of the current, changing from slow activation through Na_V_1.8 channels to a faster one that flows through Na_V_1.3/Na_V_β_3_ channels, which would reduce the activation threshold of APs and promote high-frequency firing, thus contributing to the hyperexcitability observed in injured sensory neurons [10,52]. However, Na_V_1.3 channels have been reported to be preferentially upregulated in medium- and large-sized DRG neurons after nerve injury and may not fully compensate for the loss in the functional expression of Na_V_1.8 and Na_V_1.9 channels in small-diameter sensory neurons [33,53].

The Na_V_β_1_ subunit presents a complex scenario. Although its expression increases current density through Na_V_ channels [54], its role in neuropathic pain remains unclear. Na_V_β_1_ knockout mice die prematurely, hampering behavioral studies [55], but their DRG neurons are hyperexcitable, suggesting possible allodynia [56]. Furthermore, increased Na_V_β_1_ mRNA levels in sympathetic nerve injury models complicate determining its precise role in neuropathic pain [46].

### 2.4. Na_V_ Channels as Therapeutic Targets for Neuropathic Pain

Nerve conduction through peripheral nerves has long been blocked by inhibiting the activity of Na_V_ channels to combat pain. Studies in animals and humans have validated sodium channels, mainly Na_V_1.7, Na_V_1.8, and Na_V_1.9, as viable targets for pain treatment [57]. This is because, as already mentioned, Na_V_ channels play a crucial role in the hyperexcitability of nociceptors and, therefore, in the underlying mechanism of nerve signal conduction in neuropathic pain. However, the development of effective treatments targeting Na_V_ channels has yet to advance decisively. Most studies emphasize the importance of using selective blockers for different Na_V_ channel subtypes, which could offer pain relief and minimize side effects.

Creating specific blockers for Na_V_1.7 channels is a promising approach to treating neuropathic pain, as it may help to avoid the side effects linked to non-selective sodium channel blockers [58]. Initially, a series of drugs based on benzodiazepines developed to inhibit Na_V_1.7 channels specifically showed the inhibition of spontaneous neuronal activation in animal models. They reversed tactile allodynia in spinal nerve ligation (SNL) models. Subsequently, a series of imidazopyridine-based blockers with improved pharmacokinetics and a significantly greater efficacy in SNL models were also developed [59,60,61]. Other compounds selective for the inhibition of Na_V_1.7 channels include biphenylthiazolcarboxamides and biphenylpyrazoles, as well as ProTx-II, a peptide isolated from tarantula venom that selectively inhibits Na_V_1.7 with an approximately 100-fold selectivity over other isoforms [62,63]. However, the clinical use of these compounds has been limited by their affinity for other Na_V_ channel isoforms and their ineffectiveness in reducing pain in the short term after administration [58].

Animal models in which Na_V_1.7 has been knocked out have revealed its contribution to neuropathic pain, and several works suggest that Na_V_1.7 activity regulates endogenous opioid release, such that the combination of a Na_V_1.7 channel inhibitor with an opioid may provide synergistic analgesia with fewer side effects [64,65]. Specifically, it has been shown that a complete blockade of the sodium currents in cultured wild-type DRG neurons with TTX increased the expression of opioid peptides and that the absence of Na_V_1.7 channels in the knockout mice was associated with the upregulation of Penk, the precursor of met-enkephalin, found at high levels in the sensory neuron terminals of Na_V_1.7-null mice [64]. Therefore, the combination of Na_V_1.7 channel antagonists with enkephalinase inhibitors or low-dose opioids has shown significant analgesic effects by reducing opioid-related side effects.

Interestingly, a specific regulatory sequence within the Na_V_1.7 channel structure involved in the molecular mechanism of chronic pain was identified and proposed as a new target for therapeutic intervention to alleviate neuropathic pain [66,67,68,69]. This sequence, called the collapsin response mediator protein 2 (CRMP2) regulatory sequence (CRS), seems to be crucial for the functional coupling between Na_V_1.7 channels and CRMP2, a cytosolic protein involved in regulating sodium channel activity [66,67]. A decoy peptide corresponding to the CRS reduced Na_V_1.7 currents and the presynaptic expression of the channels, decreasing the release of calcitonin gene-related peptide (CGRP) associated with pain signaling.

More importantly, the CRS peptide effectively reversed nerve-injury-induced mechanical allodynia in rodent models without causing motor impairment or altering normal physiological pain sensation [68]. Finally, an AAV-mediated gene therapy strategy introduced a plasmid encoding the Na_V_1.7–CRS gene into sensory neurons. This approach reduced the function of Na_V_1.7 channels in animal models, decreasing mechanical allodynia associated with nerve injury and chemotherapy-induced peripheral neuropathy [68]. These findings underscore the potential of the CRS domain as a therapeutic target for the management of neuropathic pain.

The selective targeting of Na_V_1.8 channels also represents a promising strategy for treating neuropathic pain. Several compounds have been developed to selectively inhibit these channels. In particular, A-803467 and A-887826 exhibit over a 100-fold selectivity for Na_V_1.8 (IC_50_ of 8 nM) compared to other sodium channel blockers and have shown efficacy in reducing neuropathic pain in rodent models [61,70,71,72]. This selectivity reduces the risk of unwanted systemic side effects associated with non-selective Na_V_ channel blockers. Likewise, dexpramipexole, a benzothiazole-like compound, has shown a high selectivity for these channels, effectively blocking TTX-R conductance in DRG neurons with an IC_50_ of ~300 nM and analgesic effects in various pain models, including those induced by nerve injury and diabetes.

Lastly, VX-548 (suzetrigine) is a more recently discovered Na_V_1.8 channel selective inhibitor, effective in treating acute pain. By inhibiting these channels, VX-548 prevents sensory neurons from transmitting pain signals to the spinal cord and brain, significantly reducing painful sensations [73,74]. Therefore, VX-548 is a first-in-class non-opioid analgesic, approved recently by the Food and Drug Administration (FDA), for treating adult patients experiencing moderate to severe acute pain, such as pain following injury, illness, or surgery [73]. However, it is not yet approved for the management of neuropathic pain, though it is being evaluated for neuropathic pain conditions, including painful diabetic peripheral neuropathy (DPN) and painful lumbosacral radiculopathy (LSR). Data from Phase 2 and 3 trials for chronic pain (including neuropathic pain) have shown mixed results, with apparent efficacy for acute pain but unresolved questions regarding chronic pain conditions.

At the molecular level, VX-548 exhibits potent state-dependent inhibition of Na_V_1.8 channels, characterized by a “reverse use dependence” mechanism. This means that it binds tightly to the channels in their resting (closed) state, but this inhibition can only be rapidly relieved by extensive and prolonged depolarizations. Consequently, VX-548 maintains tonic inhibition of these channels under physiological conditions. This unique mechanism distinguishes VX-548 from other Na_V_ channel inhibitors and supports its consistent and selective analgesic effect [75,76].

The clinical success of VX-548 validates Na_V_1.8 channels as a viable pharmacological target for treating acute pain, confirming their central role in peripheral nociceptive signaling. Furthermore, initial data from the evaluation of VX-548 in managing pain in DPN and LSR suggest that Na_V_1.8 channels also play a relevant signaling role during the development of neuropathic pain. However, further studies are required to validate this idea.

The treatment of neuropathic pain aimed at the selective inhibition of Na_V_1.8 channels offers several advantages. These compounds provide analgesia and may improve tolerability compared to other therapies. By targeting peripherally located Na_V_ channels, these blockers may minimize the central side effects typically seen with more broad-spectrum Na_V_ channel inhibitors. Preclinical and clinical research will contribute to a better understanding of their role and effectiveness in broader pain management contexts.

On the other hand, therapy targeting the molecular mechanisms associated with neuropathic pain involving Na_V_1.9 channels is still in development. Finding compounds that may alter Na_V_1.9 currents has proven difficult [61]. This is because the expression of these channels in heterologous systems is complex and tends to run down quickly in sensory neurons [77]. An innovative strategy was developed in which individual voltage-sensor paddles from Na_V_1.9 were transplanted into chimeric constructs of voltage-gated (K_V_) channels to identify toxins that may interact with native Na_V_1.9 channels [78]. Although this study showed that Na_V_1.9 channels have a distinctive pharmacological profile and that the voltage-sensor paddles could be promising targets, it was unclear to what extent the chimeric channels reproduced the pharmacological properties of native channels.

## 3. Voltage-Gated Calcium (Ca_V_) Channels in Neuropathic Pain

### 3.1. Structure and Function of Ca_V_ Channels

Ca_V_ channels are the preferential route for the entry of calcium ions into excitable cells. These channels are activated in response to the depolarization of the plasma membrane and, thus, allow for the selective entry of calcium. In this way, Ca_V_ channels contribute to determining cell excitability. Additionally, calcium entering cells acts as a second chemical messenger that initiates and regulates multiple physiological processes, including gene expression and neurotransmitter release, among many others. Therefore, Ca_V_ channels play a dual role by linking electrical signals at the cell surface with biochemical responses within the cell [79,80,81,82].

**Figure 4:**
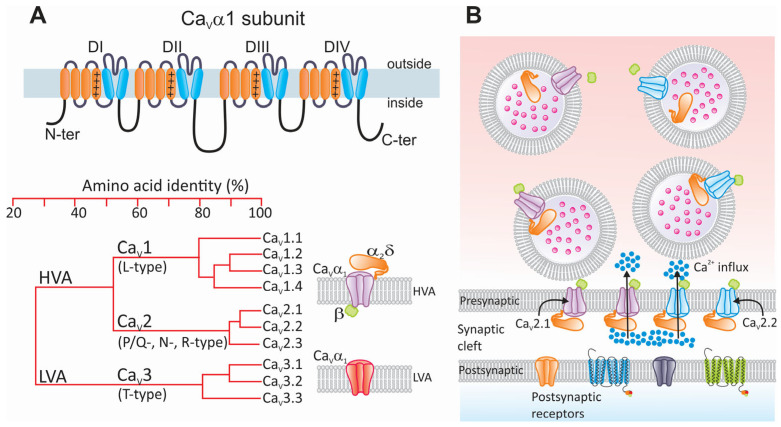
Structure and classification of Ca_V_ channels and their participation in neurotransmission. (**A**) Schematic representation of the Ca_V_α_1_ subunit illustrating its membrane topology. Like Na_V_ channels and some members of the K_V_ channel family, the main Ca_V_α_1_ subunit is a protein composed of four relatively conserved homologous repeat domains (DI–DIV) containing six α helices each (upper panel). The fourth α helix of each repeat domain contains a sequence of regularly spaced positively charged (+) basic residues that sense changes in transmembrane voltage. The loops connecting the repeat domains, as well as the amino and carboxyl termini, are intracellular. The lower left panel shows a phylogenetic tree illustrating the evolutionary relationship among members of the Ca_V_ channel family. The structural homology comparison is based on the alignment of the human channels. LVA and HVA stand for high- and low-voltage-activated, respectively. HVA channels (Ca_V_1 and Ca_V_2) are oligomeric complexes whose composition, in addition to the pore-forming Ca_V_α_1_ subunit, includes two auxiliary subunits called Ca_V_β and Ca_V_α_2_δ (shown in green and orange, respectively). On the other hand, LVA (Ca_V_3) channels function as monomers of the main Ca_V_α_1_ subunit (lower right panel). (**B**) In response to membrane depolarization caused by the arrival of an AP, presynaptic Ca_V_ channels allow for the entry of calcium ions (blue dots) from the extracellular space to the synaptic terminals. The most relevant channel subtypes involved in this event are Ca_V_2.1 and Ca_V_2.2 (shown in purple and blue, respectively). Increased intracellular calcium concentration at active sites promotes fusion of neurotransmitter-containing vesicles through the SNARE complex. Neurotransmitters (pink dots) released into the synaptic cleft diffuse until they bind to their receptors located on the postsynaptic membrane.

Based on their biophysical and pharmacological properties, voltage-gated calcium (Ca_V_) channels have been classified into T, L, N, P, Q, and R subtypes. However, the most used classification is based on the voltage range at which they apparently activate, separating them into the following two categories: low- and high-threshold channels, LVA and HVA, respectively. The T-type channel is the only low-threshold channel described, while the L-, N-, P-, Q-, and R-type channels are considered to be high-voltage-activated channels [79,80,81,82,83,84] (Figure 4A).

From a molecular perspective, LVA (Ca_V_3) channels are monomers formed solely by the main Ca_V_α_1_ subunit. HVA channels (Ca_V_1 and Ca_V_2) are more complex oligomers formed by the Ca_V_α_1_ subunit together with auxiliary subunits such as Ca_V_β and Ca_V_α_2_δ. The structure of Ca_V_α_1_ is similar to the Na_V_α subunit, consisting of four domains, each with six transmembrane segments [82,85] (Figure 4A).

Ca_V_ channels comprise a Ca_V_α_1_ ion-conducting subunit and may have associated auxiliary subunits depending on the channel subtype. The Ca_V_α_1_ subunit, in turn, consists of four homologous repeat domains, each with six transmembrane segments called S1 to S6. The S4 segment, having positively charged amino acids, can detect changes in transmembrane potential and functions as the channel’s voltage sensor. Between the S5 and S6 segments, the P segment is located, which contains the amino acids that form the ion selectivity filter. Furthermore, four isoforms of the Ca_V_β auxiliary subunit (Ca_V_β_1_ to Ca_V_β_4_) have been identified [86]. These proteins have an intracellular localization. They contribute to regulating the voltage dependence of the channels and the kinetic properties of the currents and allow the channel complex to interact with intracellular signaling molecules that modulate its activity [86,87].

Likewise, it is known that the Ca_V_α_2_δ auxiliary subunits favor the membrane expression of Ca_V_ channels (Figure 4A). Four subtypes of these proteins have been described (Ca_V_α_2_δ-1 to Ca_V_α_2_δ-4), encoded by independent genes (*CACNA2D*). These genes are initially translated into precursor proteins that are proteolytically processed, giving rise to two peptides, Ca_V_δ and Ca_V_α_2_, with the first anchored to the plasma membrane through a GPI motif and the second being completely extracellular, which remain linked by a disulfide bond [88]. The Ca_V_α_2_ peptide is highly glycosylated and contains diverse functional regions, including von Willebrand factor A (vWFA) motifs, a metal-ion-dependent adhesion site (MIDAS), and four cache regions. Similarly to the Ca_V_β subunit, the Ca_V_α_2_δ subunits promote and stabilize the expression of Ca_V_ channels on the cell surface [79,85,89].

Finally, eight Ca_V_γ subunits have been identified, which, according to phylogenic analyses, belong to a protein subfamily originating from a single gene. Biochemical and electrophysiological studies have shown the physical and functional interactions of these subunits with the Ca_V_ channel complex [90,91,92,93]. On the other hand, it is known that the Ca_V_γ_2_ subunit can also bind to proteins containing the PDZ domain and that it participates in the intracellular trafficking of the AMPA receptor [94].

In mammals, ten different Ca_V_α_1_ subunits encoded by independent genes (*CACNA1*) are expressed, which, from a molecular point of view, group Ca_V_ channels into three subfamilies [81,82]. The first (Ca_V_1) includes L-type channels with four members (Ca_V_1.1 to Ca_V_1.4). The Ca_V_2 subfamily has three members (Ca_V_2.1 to Ca_V_2.3), which give rise to currents through P/Q-type, N-type, and R-type neuronal channels, respectively. P- and Q-type channels result from the alternative splicing of the *CACNA1A* gene encoding the Ca_V_2.1α_1_ subunit [95,96].

Lastly, the Ca_V_3 subfamily groups low-activation-threshold channels and consists of three members, Ca_V_3.1 to Ca_V_3.3 (Figure 4A). These channels allow for a basal calcium influx called a window current, which helps to maintain the resting membrane potential. Likewise, since they are activated at more negative potentials than the other Ca_V_ channels, they can significantly influence cell excitability, contributing to the generation of APs and rhythmic electrical activity [79,80,81,82].

It is widely accepted that Ca_V_ channels may play a key role in the fundamental mechanisms of neuropathic pain. The contribution of these proteins to cell excitability and neurotransmission, as well as their potential role in the treatment of the condition, stresses the need to understand neuropathic pain at the cell and molecular levels [82,85,97]. The association of Ca_V_ channels with the pathogenesis of the disease occurs predominantly through the HVA channels of the P/Q- (Ca_V_2.1) and N-types (Ca_V_2.2) and the ancillary Ca_V_α_2_δ subunit. However, it has also been reported that the LVA channels of the Ca_V_3.2 class may also contribute significantly to the pathophysiology of the condition [98,99,100].

As mentioned above, the entry of calcium ions in response to the activation of Ca_V_2 channels determines the release of neurotransmitters. The calcium that enters the nerve terminal promotes the assembly of a subset of scaffolding proteins essential for anchoring synaptic vesicles containing neurotransmitters to the cell membrane and their eventual fusion [87,101,102] (Figure 4B). Therefore, alterations in the functional expression of Ca_V_2 channels will alter synaptic transmission and consequently may affect pain signaling.

### 3.2. Role of Different Ca_V_2 Channel Subunits in Nociceptive Pathways and Neuropathic Pain

Ca_V_ channels regulate neuronal excitability, synaptic transmission, and pain signaling. As we will see below, there are three subtypes of Ca_V_2 channels. Among them, Ca_V_2.1 (P/Q-type) and Ca_V_2.2 (N-type) are particularly important in neurotransmission between primary afferent fibers and neurons of the SC’s dorsal horn.

**Figure 5:**
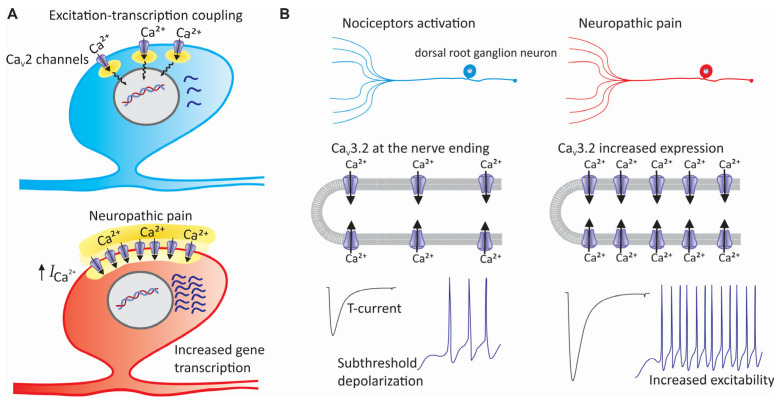
Contribution of Ca_V_ channels to the pathogenesis of neuropathic pain. (**A**) In addition to its effects on the release of neurotransmitters, alteration in the expression of Ca_V_2 channels participates in the pathophysiology of neuropathic pain by affecting the excitation–transcription coupling. This fundamental cell process links electrical activity in excitable cells to gene transcription. This implies that the calcium, once inside the cells, can activate transcription factors, either directly or through protein kinases and second messengers that control the activity of these factors. (**B**) Overexpression of Ca_V_3 channels in sensory neurons during neuropathic pain increases their excitability, decreases the firing threshold of afferent fibers, and favors repetitive firing.

Ca_V_2 channels contribute to the onset and maintenance of neuropathic pain through different cellular and molecular processes. Firstly, these channels are decisive in synaptic transmission, since their activation gives rise to transient increases in the concentration of intracellular calcium in the nerve terminals, which favors the release of neurotransmitters. In pathological conditions, however, synaptic transmission in sensory neurons may be altered, increasing the release of chemical pain mediators such as glutamate and substance P and inducing central sensitization, one of the main features of neuropathic pain. Second, the activity of Ca_V_2 channels can be regulated by phosphorylation and other post-translational modifications, which may promote their functional expression during neuropathic pain. Finally, transient increases in intracellular calcium can cause, in pathological conditions, changes in gene expression patterns that promote the activation of transcription factors that target genes associated with chronic pain [103] (Figure 5A).

The contribution of Ca_V_2.1 (P/Q-type) channels to pain perception is being studied in detail and is beginning to be revealed. These channels are crucial for neurotransmitter release in the CNS, and pharmacological studies suggest their involvement in the pathophysiology of pain. While initial studies showed that intrathecal injections of specific blockers, such as AgaIVA, did not have an apparent effect on Ca_V_2.1 channels in neuropathic pain [104,105,106], subsequent research has indicated that they may influence descending pathways that modulate pain transmission in some areas of the brain [107,108]. Likewise, studies conducted in Ca_V_2.1α_1_ subunit knockout mice have revealed alterations in their response to nociceptive stimuli [109]. However, eliminating the pore-forming subunit of Ca_V_2.1 channels does not significantly modify the response to thermal stimuli [109].

Ca_V_2.2 channels are predominantly expressed in the presynaptic nerve terminals of central and peripheral neurons [110]. They are crucial for releasing neurotransmitters relevant to generating pain signals, such as glutamate and GABA. In addition, the activity of these channels is modulated by the activation of various G protein-coupled receptors (GPCRs) involved in nociception, including opioid, cannabinoid, neuropeptide Y, and substance P receptors [111,112]. Research has shown a significant relationship between the knockout of Ca_V_2.2 channels and neuropathic pain, consistent with their contribution to the molecular pathophysiology of this condition. Multiple studies suggest that the genetic ablation of these channels can result in reduced pain responses across various models of neuropathic pain [113,114]. The absence of Ca_V_2.2 channels affects the release of substance P and CGRP, and this reduction in neurotransmitter release contributes to a decrease in overall pain signaling.

Ca_V_2.2 channels are concentrated in nerve terminals located in laminae I and II of the dorsal horn of the spinal cord, where they transmit pain signals arriving on C and Aδ fibers. Interestingly, these channels in primary afferent fibers contribute to developing allodynia and hyperalgesia after nerve injury [115], as discussed below.

Several studies have shown that mutually exclusive splicing patterns in the gene encoding the Ca_V_2.2α_1_ subunit modulate the function of N-type channels in sensory neurons and can influence pain transmission. In particular, an exon 37a-containing isoform whose expression is restricted to DRGs correlates closely with significantly larger N-type currents in nociceptive neurons [116,117]. The preferential inclusion of exon 37a in sensory neurons generates a module in the C-terminus of the Ca_V_2.2α_1_ subunit that mediates channel inhibition in a voltage-independent manner, which requires tyrosine kinase activation [118,119]. Furthermore, exon 37a enhances the μ-opioid-receptor-mediated inhibition of N-type channels [120], contributing to defining the molecular nature of the voltage-independent inhibition of N-type channels in the pain pathway.

Likewise, research in animal pain models has shown that the expression of the Ca_V_2.2α_1_ subunit is significantly increased [100]. Mice subjected to partial sciatic nerve ligation showed an increased current amplitude through N-type channels and increased mRNA levels for the gene encoding the Ca_V_2.2α_1_ protein in DRG neurons [121,122]. Likewise, in a chronic constrictive nerve injury model, the Ca_V_2.2α_1_ protein was upregulated in lamina II of the SC dorsal horn [123]. Furthermore, in nerve ligation models, a significant increase in the expression of Ca_V_2.2 channels with a subsequent increase in the amplitude of the current was reported, which further facilitated the excitatory synaptic transmission of Aδ and C fibers in the SC dorsal horn [122].

Research on the cellular and molecular bases of neuropathic pain has shown that ubiquitination may contribute to its establishment and maintenance by regulating the turnover of synaptic proteins. Specifically, it has been described that the active zone protein RIM1α participates in the development of the condition by binding and positively regulating the expression of Ca_V_2.2 channels. It is also known that RIM1α-associated spinal allodynia is mediated by Fbxo3, a protein that reduces the Fbxl2-dependent ubiquitination of RIM1α. When deubiquitinated, RIM1α can bind directly to these channels, increasing its expression in the nerve terminals of the dorsal horn of the SC [124].

The activation of nociceptin opioid peptide (NOP) receptors, also known as opioid-like receptor 1 (ORL-1), results in the G protein-dependent regulation of Ca_V_2.2 channels [125,126,127]. This results in a decreased current amplitude, with consequent alterations in presynaptic calcium levels and impairment in neurotransmission [128]. Due to the widespread expression of both NOP and Ca_V_2.2 channels in the brain, dorsal horn of the SC, and DRG, alterations in this system result in different neurological conditions, including neuropathic pain. As mentioned, Ca_V_2.2 channels are crucial for pain processing by controlling the synaptic strength on C and Aδ afferent fibers. Thus, reducing calcium influx by activating NOP receptors may decrease the release of CGRP and substance P, neurotransmitters involved in pain signaling. This is beneficial in neuropathic pain, where the expression of Ca_V_2.2 channels is generally upregulated [126,129].

### 3.3. The Role of the Ca_V_α_2_δ-1 Auxiliary Subunit in Neuropathic Pain

The α_2_δ subunits of Ca_V_2 channels have been shown to play crucial roles in nociceptive signaling [130,131,132,133]. As mentioned earlier, these proteins are essential in the function and regulation of these channels by contributing to the intracellular trafficking, voltage dependence, and kinetics of the currents [134,135,136,137,138]. In particular, the Ca_V_α_2_δ-1 subunit, which is expressed in excitable cells, including neurons, is essential for presynaptic functions such as synapse formation, the regulation of synaptic plasticity, and the control of the calcium concentration in the synaptic cleft [138,139,140]. The protein contains several functional regions that allow for interactions with the channel complex and other synaptic molecules. Research on the structure of Ca_V_α_2_δ has identified a von Willebrand factor A (VWA) region along with four cache domains. The VWA region is critical for interaction with the Ca_V_α_1_ subunit [138].

It has been reported that Ca_V_α_2_δ expression may increase at both the mRNA and protein levels in sensory neurons after spinal nerve ligation and in animal models of diabetic neuropathy [130,141,142,143,144,145]. This change is accompanied by AP discharges in the injured neurons due to an increase in the functional expression of Ca_V_2.2 channels mediated by the exacerbated expression of the Ca_V_α_2_δ subunit. Consistent with this, genetic ablation of the Ca_V_α_2_δ-1 subunit significantly decreased the expression of Ca_V_2.2 channels on the cell membrane of DRG neurons and in the dorsal horn of the SC [146]. This alteration in neuronal excitability can affect the release of neurotransmitters associated with neuropathic pain pathways [122]. Interestingly, mice overexpressing Ca_V_α_2_δ-1 exhibit neuropathic pain symptoms without nerve damage, whereas Ca_V_α_2_δ-1-deficient mice show deficits in sensitivity after nerve injury [147,148,149].

Several studies support an important role of the interaction between the Ca_V_α_2_δ-1 subunit and thrombospondin-4 (TSP4), a glycoprotein found in the extracellular matrix, in nerve-injury-induced neuropathic pain, mediated through aberrant excitatory synapse formation and presynaptic neurotransmission in the SC [150,151,152]. Peripheral nerve injury induces the upregulation of both proteins in the SC that precedes the onset and correlates with the duration of neuropathic pain [130,141,142,143,153,154,155]. The inhibition of this regulation or the genetic ablation of Ca_V_α_2_δ-1 or TSP4 prevent the onset and development of the disease [152,155,156]. The mechanism by which an exaggerated expression of TSP4 alters the function of Ca_V_ channels remains to be established. In this regard, it has been reported that TSP4 can differently affect the distinct types of channels in sensory neurons, decreasing the currents passing through HVA channels and increasing those flowing through LVA channels [154], which is paradoxical given that, unlike what occurs with HVA channels, a clear role for auxiliary subunits, including Ca_V_α_2_δ-1, in the functional expression of LVA channels has not yet been established. Further research is required to better understand the origin of this discrepancy.

Similarly, research has demonstrated that the Ca_V_α_2_δ-1 subunit interacts with N-methyl-D-aspartate receptors (NMDARs) to create a complex that increases their activity by promoting their trafficking to synapses [157,158]. NMDARs are preferentially expressed in postsynaptic neurons, but are also present in presynaptic neurons, influencing neurotransmitter release and synaptic plasticity [159]. The activation of presynaptic NMDARs leads to increased calcium and the exocytosis of secretory vesicles, resulting in greater glutamate release. Under normal conditions, NMDARs are inactive; however, these receptors become tonically active in neuropathic pain [160]. Notably, models of neuropathic pain have shown an elevated expression of Ca_V_α_2_δ-1/NMDAR complexes, indicating their potential involvement in pain mechanisms [149].

### 3.4. Contribution of the Ca_V_3.2 Channels to Neuropathic Pain

On the other hand, presynaptic T-type calcium channels play a crucial role in nociceptive signaling, and their dysregulation can lead to the development of neuropathic pain [97,98,99]. Notably, the Ca_V_3.2 isoform, expressed in primary afferent neurons, spinal dorsal horn neurons, and supraspinal brain regions, is particularly important in processing pain signals.

It is known that Ca_V_3.2 channels participate in the regulation of neuronal excitability. Their activation lowers the threshold for APs, which can alter pain signaling under pathological conditions [97,161,162]. An increased expression of these channels in DRG neurons has been linked to heightened neuronal firing and chronic pain (Figure 5B). In contrast, silencing Ca_V_3.2 channels in DRG neurons using antisense oligonucleotides or siRNA significantly reduces mechanical nociception and tactile allodynia [163,164]. In addition, experimental evidence indicates that antagonists targeting these channels may reduce neuronal excitability and provide analgesia in models of neuropathic pain [99]. However, beyond peripheral and spinal mechanisms, Ca_V_3.2 channels may also play an important role in specific brain areas, contributing to pain perception and modulation [161,162]. Interestingly, the inhibition of these channels in the brain has also been found to have analgesic effects.

Likewise, experimental evidence shows that although the total expression of Ca_V_3.2 in DRG neurons may increase in neuropathic pain models, the membrane expression of these channels is significantly augmented without changes in total expression [99,165]. Additionally, the accumulation of Ca_V_3.2 in uninjured nerves may contribute to neuropathic pain due to interactions with injured axons, mediated by increased levels of NGF and tumor necrosis factor-α (TNF-α), which regulate T-type calcium channels [166]. In the early stages of chronic pain, an increase in Ca_V_3.2 expression is favored by the transcription factor Egr-1 [167].

Phosphorylation, ubiquitination, and other post-translational modifications are known to contribute to the development of neuropathic pain after nerve injury [97,99,168]. In this regard, it has also been documented that, after spinal nerve injury, there is an upregulation in the expression of Ca_V_3.2 channels and the Cdk5 kinase in DRG and SC dorsal horn neurons, which is associated with mechanical allodynia. Cdk5 directly phosphorylates Ca_V_3.2 channels, increasing their functional expression and enhancing neuronal excitability, contributing to neuropathic pain. In contrast, the inhibition of Cdk5 decreases the firing of compound APs in spinal nerves and modifies the paw withdrawal threshold in animals with allodynia induced by spinal nerve ligation (SLN) [169]. Interestingly, the study of functional and cellular localization changes in Ca_V_3.2, as well as Ca_V_2.2 channels and Cdk5 within intact L3-4 afferent fibers adjacent to the injured peripheral nerve at L5-6, show that both the channels and the kinase are altered in intact neurons after injury, evidencing an additional molecular mechanism underlying neuropathic pain. Furthermore, nerve injury at L5-6 has been shown to modify the slow and fast components of compound APs recorded in the L4 dorsal root, and these changes may be mediated by the effects of Cdk5 on Ca_V_ channel function and localization [169].

Finally, it is worth mentioning that the ubiquitin–proteasome system also regulates Ca_V_3.2 channels, influencing their expression and functional activity in neuropathic pain [99]. Research shows that USP5 is a deubiquitinating enzyme that decreases the ubiquitination of Ca_V_3.2 channels during neuropathic pain [167,170]. This action prevents the channels from being internalized and increases their presence on the cell membrane, leading to higher T-type calcium currents and pain sensitivity. In the early stages of neuropathic pain, the transcription factor Egr-1 controls the expression of Ca_V_3.2 channels, while in later stages, USP5 is responsible for further increasing Ca_V_3.2 expression. Studies have shown that knocking down USP5 results in an increased ubiquitination of Ca_V_3.2, decreased protein levels of the channel, and reduced whole-cell currents [170,171,172]. Conversely, increasing USP5 activity leads to greater activity of Ca_V_3.2 channels in models of neuropathic pain.

### 3.5. Ca_V_ Channels as Potential Therapeutic Targets for Neuropathic Pain

Gaining insight into the function and potential of Ca_V_ channels as therapeutic targets has offered valuable information for managing chronic pain conditions. For instance, Ca_V_2.2 channels are key in pain signaling because they help release neurotransmitters from sensory neurons. Blocking these channels can prevent the release of neuropeptides that transmit pain, making them a promising target for treating neuropathic pain.

Several studies stress significant advancements in studying Ca_V_2.2 channels for treating neuropathic pain. Ziconotide, a synthetic version of the marine peptide ω-conotoxin MVIIA, has been established as an effective blocker of these channels for treating severe chronic pain [111,173]. It is administered intrathecally and has shown efficacy in several neuropathic pain models. The mechanism of ziconotide involves blocking calcium entry, which is crucial for releasing the neuropeptides substance P and CGRP in sensory neurons [174].

Furthermore, in animal models, treatment with ziconotide can prevent hyperalgesia and allodynia, confirming the role of Ca_V_2.2 channels in establishing neuropathic pain. Ziconotide is about ten times more potent than intrathecally administered morphine [175]. However, its clinical use is limited by side effects [111]. Leconotide is a newer blocker of Ca_V_2.2 channels, which has emerged as an alternative to ziconotide. This compound has been shown to have antihyperalgesic effects and offers a better side effect profile [176]. On the other hand, an alternative for pain relief has focused on developing small molecules that function as inhibitors of Ca_V_2.2 channel activity. These molecules aim to provide similar benefits to ziconotide and leconotide without the disadvantages associated with peptide administration. Some of these peptides have been designed to disrupt the coupling of the main subunit Ca_V_α_1_ with other intrinsic or extrinsic proteins in the channel complex. In this context, various studies suggest that collapsin response mediator protein 2 (CRMP-2) is an important molecular interactor of Ca_V_2.2, regulating its function and, consequently, the release of neurotransmitters in sensory neurons. The overexpression of CRMP-2 increases the current density through Ca_V_2.2 channels and enhances the release of CGRP, which participates in pain transmission.

It has also been reported that disrupting the CRMP-2/Ca_V_2.2 complex with specific peptides, such as TAT-CBD3, Ct-dis, and R9-CBD3-A6K, reduces the excessive neurotransmitter release associated with chronic pain, showing antinociceptive effects in neuropathic pain models [177,178,179,180]. Moreover, the potential of quinazolines and benzoylpyrazolines as agents that disrupt the coupling between Ca_V_α_1_ and Ca_V_β subunits has been investigated. These compounds have shown the ability to decrease currents through Ca_V_2.2 channels, alter their presynaptic localization, and inhibit the release of CGRP, exhibiting antinociceptive properties in various pain models, including neuropathic pain [181].

Likewise, Khanna and his colleagues also showed that the inhibition of the interaction between Ca_V_α_1_ and Ca_V_β subunits reduces the excitability of DRG neurons, leading to a decrease in acute and neuropathic pain in several animal models [182]. Specifically, the authors developed a molecule identified as IPPQ that selectively binds to Ca_V_β, inhibiting its coupling with the Ca_V_α_1_ subunit of Ca_V_2.2 channels. This leads to the delocalization of presynaptic channels, a decrease in the amplitude of calcium currents in sensory neurons, and a reduction in the release of the nociceptive neurotransmitter CGRP in the SC. This same research group recently designed a small peptidomimetic molecule derived from the CRMP2 peptide called CBD3063. This compound selectively inhibits the interaction between Ca_V_2.2 channels and CRMP2, reducing calcium entry and neurotransmitters’ release linked to pain signaling. Additionally, CBD3063 showed efficacy in animal models by reversing neuropathic pain without affecting sensory or cognitive functions, suggesting a favorable side effect profile [183].

Likewise, C2230 is a novel use- and state-dependent blocker of Ca_V_2.2 channels, recently reported for its potential as an analgesic across various pain models [184]. It effectively reverses pain behaviors associated with neuropathic pain without negatively impacting motor or cardiovascular functions. The compound stabilizes Ca_V_2.2 channels in the inactivated state, resulting in use-dependent inhibition during high-frequency stimulation. This reduces calcium influx in sensory neurons, significantly decreasing excitatory postsynaptic currents and neurotransmitter release in the SC. Additionally, C2230 lowers calcium responses in the parabrachial nucleus, a critical area for pain processing, and alleviates adverse reactions to mechanical stimuli following neuropathic injury [184].

Recent research has highlighted the role of Ca_V_α_2_δ-1 auxiliary subunit ligands as targets for treating neuropathic pain. Gabapentin (GBP) and pregabalin, first- and second-generation Ca_V_α_2_δ-1 ligands, have been shown to effectively alleviate the symptoms of this condition [185,186,187]. These drugs work by preferentially binding to the Ca_V_α_2_δ-1 and Ca_V_α_2_δ-2 subunits of Ca_V_ channels [188], modulating calcium influx, and reducing the release of excitatory neurotransmitters, which contributes to their analgesic effects [189,190].

GBP has been shown to inhibit calcium entry through Ca_V_ channels by acting directly on the Ca_V_α_2_δ subunit [188]. Findings suggest that GBP’s ability to downregulate Ca_V_2.2 channels contributes to its analgesic effects, particularly in the management of neuropathic pain, by reducing the release of neurotransmitters at nerve terminals. GBP’s mechanism of action was initially proposed by our research group and is based on the premise that the drug enters cells through an L amino acid transport system and interacts with the subunit of the channels inside the cell, disrupting (or preventing) the association of Ca_V_α_2_δ-1 with the channel complex. This results in inadequate trafficking of the channels to the plasma membrane [191].

Subsequent studies provided experimental support for this mechanism, demonstrating that the VWA domain of Ca_V_α_2_δ subunits plays a critical role in the intracellular trafficking of Ca_V_ channels. Furthermore, these studies revealed that interaction with GBP can disrupt the normal functioning of this domain [192]. Additionally, it has been found that pregabalin can affect the trafficking of Ca_V_α_2_δ-1 to the presynaptic terminals of DRG neurons, likely using a mechanism similar to that of GBP, thereby reducing calcium entry and neurotransmitter release in the SC [144]. Finally, it has also been shown that some derivatives of γ-aminobutyric acid (GABA), which exhibit anticonvulsant and antinociceptive properties, relate to their ability to bind to the Ca_V_α_2_δ subunit of Ca_V_2.2 channels, similar to the mechanism observed with GBP and pregabalin [193,194].

The novel Ca_V_α_2_δ ligand mirogabalin represents a third-generation option aimed explicitly at peripheral neuropathic pain and shows promising clinical applications [195,196]. NVA1309, another next-generation ligand, may offer a greater efficacy with fewer side effects than existing treatments [195,197]. NVA1309 is a gabapentinoid that binds to the Ca_V_α_2_δ-1 subunit at a specific site (R243), which is also utilized by other gabapentinoids like pregabalin. NVA1309 and mirogabalin inhibit Ca_V_2.2 currents in vitro and reduce Ca_V_2.2 expression in the plasma membrane more effectively than pregabalin [195].

Therefore, auxiliary subunits of calcium channels, particularly Ca_V_α_2_δ, represent promising targets for treating neuropathic pain due to their significant role in regulating calcium channel activity and neuronal excitability. By targeting the auxiliary subunits instead of the main Ca_V_α_1_ channel subunit, therapies with fewer side effects than traditional channel blockers can be created. This approach may lead to treatment options that are better tolerated by patients with chronic pain.

Ca_V_3 channel blockers, such as ethosuximide, have shown the ability to improve symptoms of neuropathic pain in animals with nerve injury. However, clinical studies have been inconclusive due to their low patient effectiveness. Similar observations have been made with small molecules that block Ca_V_3.2 channels, such as ABT-639, TTA-P2, TTA-A2, and Z944, which have not produced relevant results despite their promising start in preclinical studies [99,198,199,200,201,202]. Additionally, cannabinoids have shown effectiveness in alleviating neuropathic pain by inhibiting Ca_V_3 channels and increasing potassium currents through BK channels. An intrathecal injection of the CB1/CB2 receptor agonist NMP-7 has been shown to inhibit neuropathic pain induced by nerve injury in animal models through mechanisms involving CB2 receptors and Ca_V_3.2 channels [201,203].

Interestingly, some natural compounds have shown the ability to alleviate inflammatory and neuropathic pain through their dual inhibitory action on Ca_V_ channels. Specifically, recent studies have documented that Icariside II, a prenyl-flavonol derived from the traditional Chinese herb epimedium, may have a beneficial effect on neuropathic pain by inhibiting Ca_V_3 channels and disrupting the interaction between USP5 and Ca_V_3.2 channels [204]. Furthermore, Icariside II reduces the excitability of sensory neurons and, in models of neuropathic pain, significantly decreases mechanical allodynia and thermal hyperalgesia, indicating its analgesic potential [204].

Likewise, a novel therapeutic strategy for neuropathic pain involves the synthetic peptide RD2, which selectively inhibits Ca_V_2.2 channels when administered orally. RD2 competes with ziconotide for binding to these channels at nanomolar concentrations, demonstrating a high selectivity compared to other Ca_V_ channel subtypes. In preclinical studies, RD2 significantly alleviated mechanical allodynia in rats with sciatic nerve inflammatory neuritis [205]. Unlike ziconotide, which requires intrathecal delivery and is associated with logistical challenges and side effects, RD2 offers the advantage of oral administration. However, despite its promising selectivity in vitro, RD2’s long-term effects on other systems remain uncertain, as Ca_V_2.2 channels are implicated in essential physiological processes like neurotransmission. Furthermore, efficacy data are currently limited to rodent models, with no evidence yet from primate or human studies. These factors highlight ongoing challenges in ensuring the safety and specificity of RD2 for clinical applications.

Recently, Colecraft’s research group developed an ingenious maneuver that could increase the arsenal of molecular tools available for treating neuropathic pain. Specifically, these authors showed that the targeted ubiquitination of Ca_V_ channels in sensory neurons may reduce neuropathic pain [206,207]. The system selectively ubiquitinated Ca_V_ channels in sensory neurons, reducing their number at the cell membrane and decreasing current density. The Ca_V_-aβlator system consists of a molecule designed to post-translationally reduce the number of HVA-type Ca_V_ channels in the membrane by specifically targeting Ca_V_β accessory subunits. It comprises a nanobody that indiscriminately binds to all four Ca_V_β isoforms and is fused to the HECT catalytic domain of the E3 ubiquitin ligase Nedd4L. This fusion promotes the ubiquitination of both Ca_V_α_1_ and Ca_V_β subunits, removing the channel complex from the cell membrane, thus resulting in the potent inhibition of calcium currents in various cell types [206,207].

This targeted reduction in the functional expression of Ca_V_ channels decreases neuropathic-pain-associated behaviors in animal models, showing a potential new avenue for pain therapy [207,208]. This study provides evidence that manipulating the post-translational modification, such as ubiquitination, of voltage-gated ion channels may be an effective strategy for controlling neuropathic pain, providing an alternative to current pharmacological approaches, which often have significant side effects or a limited efficacy.

The Ca_V_-aβlator strategy acts as a potent and selective inhibitor that reduces the number of Ca_V_ channels on the cell surface, especially Ca_V_2.2 (N-type), which are key for neurotransmitter release in pain pathways and an essential target for treating neuropathic pain. This is achieved through the targeted ubiquitination of Ca_V_β subunits, resulting in channel internalization and degradation. Thus, Ca_V_-aβlator enables the precise control of these calcium channel functions and holds significant therapeutic potential for cardiovascular and neurological diseases, where these channels play a pivotal role.

## 4. Voltage-Gated Potassium (K_V_) Channels in Neuropathic Pain

### 4.1. Structure and Function of K_V_ Channels

**Figure 6:**
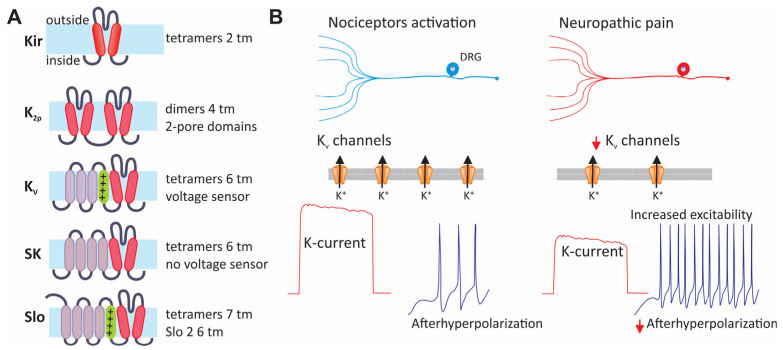
Classification and contribution of K_V_ channels to neuropathic pain. (**A**) The family of potassium channels arranged according to the structure of their main α subunits. The members of this family can be grouped into those formed by tetramers of two (Kir) or dimers of four (two pores) transmembrane segments. Likewise, channels formed by six transmembrane segments, the predominant voltage-sensitive potassium channels, assemble into a tetramer to form a functional channel. The same is also valid for the small-conductance calcium-activated potassium (SK) channels and the large-conductance channels activated by both changes in intracellular calcium and membrane voltage. (**B**) The expression of K_V_ channels is often decreased in neuropathic pain. This decreases the outward current, which usually helps to stabilize the membrane potential and opposes excitatory signals. The decreased activity of K_V_ channels during neuropathic pain may cause an increase in neuronal excitability, affecting the frequency and duration of APs.

K_V_ channels are classified into three major structural families (Figure 6A). Members of the first family correspond to the inward rectifier (Kir) channels that follow the structural pattern of the KcsA channel. This primitive channel consists of a tetramer formed by four identical subunits containing two transmembrane domains connected by a pore region, where the ion selectivity filter resides [209,210].

In mammals, Kir channels are encoded by 15 genes grouped into seven subfamilies [211]. On the other hand, members of the second family of potassium channels are formed by two pores (K2p) and four transmembrane segments; unlike the other families, their subunits assemble as dimers. Fifteen genes of this family have been found in mammals [211,212,213]. The third family of potassium channels comprises six transmembrane segments and a single pore-forming domain for ion conduction (α-subunit), including the subfamily of voltage-gated channels (K_V_1 to K_V_4). The K_V_1 subfamily is the largest, with eight different genes. Like Na_V_ and Ca_V_ channels, these channels contain a voltage sensor domain, where the fourth segment (S4) contains an array of positively charged amino acids that function as voltage-sensing elements. This also includes the K_V_7 (KCNQ), K_V_10 (ether-a-go-go), K_V_11 (erg), and K_V_12.2 (elk) subfamilies [211] (Figure 6A).

Interestingly, the K_V_5, K_V_6, K_V_8, and K_V_9 channels do not form functional channels even though they share the same general structure as the other members of the K_V_ family. For this reason, these proteins have been called silent subunits (K_V_S). However, by forming heterotetrameric channels with the K_V_2α and K_V_3α subunits, they can modulate their biophysical properties and inhibit their functional expression [211,214]. This family of six transmembrane segments includes the small-conductance calcium-activated potassium channels (SKCa) and the Slo channel subfamily [215]. Though the structure of Slo channels is similar to that of K_V_ channels, the α subunits of Slo1 and Slo3 have seven transmembrane domains instead of six. Furthermore, the α subunits of Slo channels have a large C-terminal domain [216].

K_V_ channels participate in multiple functions and are expressed in all eukaryotic cells. These channels determine the resting membrane potential in most cells and are fundamental components of the electrical activity of the cell membrane in virtually all tissues. Additionally, they help to determine the shape, duration, and frequency of APs in excitable cells. The function of voltage-gated potassium channels (K_V_) in excitable cells can often be inferred from their subunit composition, which determines their biophysical properties and interactions with second messengers, as well as their spatial and temporal expression and regulation in pathophysiological processes [211,217].

### 4.2. The Role of K_V_ Channels in Neuropathic Pain

Research on mutant mice lacking specific subunits of K_V_ channels has highlighted their direct role in nociceptive circuits, enhancing our understanding of K_V_ channel subunits in healthy and diseased conditions [217,218]. K_V_ channels typically counteract membrane depolarization that activates Na_V_ and Ca_V_ channels, thereby reducing the excitability of sensory neurons. However, a decrease in K_V_ channel activity is associated with hyperexcitability in various pain syndromes, including traumatic injuries and painful diabetic neuropathy (Figure 6B). The K_V_1.1/K_V_1.2 subunits influence the AP threshold and firing frequency in primary afferent fibers and can be affected by nerve injuries [217]. In contrast, the K_V_2.1/K_V_2.2 subunits are recruited slower, primarily impacting repolarization and AP firing frequency. High-threshold K_V_3 channels limit AP duration and neurotransmitter release at central terminals. Moreover, the downregulation of K_V_4.3 channels after peripheral axotomy contributes to mechanical hypersensitivity. At the same time, inflammatory mediators in chronic pain states can modify the functional expression of K_V_7.2/K_V_7.3 channels, increasing the excitability of DRG neurons [217,218].

Interestingly, the K_V_9.1 subunit has been postulated as a predictor of neuropathic pain. Studies showing the downregulation of its expression after nerve injury have confirmed its relevance [217,218,219]. K_V_9.1 belongs to the K_V_S subfamily, which only conducts currents with other subunits like K_V_2.1, a high-threshold channel distinguished by its slow activation and inactivation kinetics [220]. These characteristics suggest that its effect becomes more significant in the later phases of the AP and is enhanced by prolonged stimulation.

Spinal nerve ligation reduces the expression of K_V_1.2 channels in DRG neurons, presumably by affecting the expression of the enzyme Tet methylcytosine dioxygenase 1 (TET1). In line with this, the overexpression of the enzyme rescues the deficient expression of the channels by reducing methylation in the promoter of the *KCNA2* gene [221]. Furthermore, histone deacetylase 2 (HDAC2) controlled the expression of K_V_1.2 in sensory neurons in an animal model of chronic nerve constriction [222]. Finally, the microRNA miR-137 is known to regulate the function of K_V_1.2 channels, while its silencing rescues the expression and function of these channels, reducing allodynia in neuropathic animals [223].

Although spinal nerve ligation reduces mRNA levels for K_V_1.2 [224,225], changes in the functional expression of these channels could reflect post-translational processes such as phosphorylation or intracellular trafficking, independent of changes in gene expression [224]. This idea is supported by the observation that currents passing through delayed rectifier potassium channels are reduced in DRG neurons without being accompanied by changes in mRNA levels for K_V_1 and K_V_2 channels [226].

A proposed mechanism for the involvement of K_V_2.1 channels in neuropathic pain is decreased functional expression. Indeed, research has found reductions in neuronal K_V_2.1 expression following nerve damage [218,227,228,229]. On the other hand, electrophysiological recordings in DRG neurons have shown that the inhibition of K_V_2.1 channels affects posthyperpolarization following the firing of APs, modifying the refractory period between spikes. Therefore, it has been postulated that, in neuropathic pain, alterations in the expression of these channels may affect AP propagation along the axon during repeated firing [218,219,227,229]. Indeed, the excitability of sensory neurons increases after a blockade of K_V_2.1 channels, allowing for a higher firing rate. This suggests that K_V_2.1 channels may function as a brake on neuronal excitability, which is significantly altered under neuropathic pain conditions.

An alternative possibility to reduce the activity of K_V_2.1 channels that occurs in neuropathic pain is through the deregulation of K_V_S subunits, such as K_V_9.1, which participates in the formation of the functional tetrameric channel [227,230]; K_V_9.1 is the only K_V_S subunit implicated in the pathophysiology of neuropathic pain. In animal models, it shows a significant and rapid downregulation in DRGs after nerve injury, which correlates with the presence of the pain phenotype [218,227]. Likewise, the inhibition of K_V_9.1 with intrathecally applied siRNAs recapitulates mechanical allodynia phenotypes. K_V_9.1 silencing results in lower firing thresholds and shortening after hyperpolarization in DRG neurons, a phenotype reminiscent of the inhibition of K_V_2.1 channels [218]. These data suggest that the alteration in K_V_2.1 channel activity during neuropathic pain signaling is due, at least in part, to a loss of its functional interaction with K_V_9.1.

K_V_4 channels are also expressed in sensory neurons of the DRG [231]. Electrophysiological and molecular studies using antisense probes implicate the K_V_4.1 and K_V_4.3 channels as the molecular correlates of A-type potassium currents in DRG neurons [232]. The function and expression of these channels are regulated by various signaling pathways, accessory subunits such as K_V_4-channel-interacting proteins (KChIPs), and transcription factors such as restrictive neuron silencing factor (REST) [233,234], which, interestingly, suppresses the transcription of the *KCND3* gene encoding K_V_4.3 channels after nerve injury [235]. The downregulation of mRNA for K_V_4.3 channels and their membrane expression occurs in DRG neurons in several nerve injury models, implicating the dysfunction of these channels in neuropathic pain [217].

Furthermore, the regulatory subunits KChIP1, KChIP2, and DPP10 form a complex with K_V_4.3 channels in DRG neurons, and spinal nerve ligation is known to downregulate the expression of components of this molecular complex. Conversely, the overexpression of K_V_4.3, KChIP1, and DPP10 is accompanied by the attenuation of nerve-ligation-induced mechanical hypersensitivity and the partial recovery of membrane levels of the complex members in injured DRGs. These data show that potassium channel modulatory subunits participate in developing K_V_4.3-mediated neuropathic pain [236].

Likewise, a current called M that flows through K_V_ channels also contributes to the regulation of pain pathways. This low-threshold non-inactivating current is regulated by the activation of muscarinic receptors. The M current, like other potassium currents, regulates membrane potential and, consequently, neuronal excitability [218,237,238]. This current arises from the activity of homomeric K_V_7.2 channels, also known as KCNQ2, or heteromeric channels formed by the combination of K_V_7.2 and K_V_7.3, also called KCNQ3 [239,240,241]. It is also known that sensory neurons in the DRG express both types of channels, and that they also display M currents upon electrophysiological examination [242].

Furthermore, it has been reported that the expression of the K_V_7.2 and K_V_7.3 channels decreases in neuropathic pain models due to nerve constriction, and that, in consequence, the M current is reduced in sensory neurons of the DRG [243,244]. Molecular studies on the mechanisms associated with the downregulation of these channels in response to nerve injury show that the genes encoding K_V_7 channels have binding sites for repressor element 1 (NRSE) and that the effects of nerve ligation are linked to the transcription factor REST, whose expression increases in sensory neurons in response to nerve injury [244,245,246].

### 4.3. K_V_ Channels in Neuropathic Pain Therapy

Although K_V_1.2 channels have been identified as molecular actors in the pathophysiology of neuropathic pain as regulators of neuronal excitability, they have not yet been used as a therapeutic target. However, there is experimental evidence that restoring normal levels of K_V_1.2 in DRGs and dorsal horn of the SC decreases pain in neuropathic animals, indicating its potential as a therapeutic target [223]. Similarly, K_V_4 channels have not yet been targeted for clinical treatments for neuropathic pain. Still, they are considered as promising candidates for the future development of analgesic drugs because, in experimental studies, rescuing the negative regulation of these channels has shown potential to relieve pain in animal models.

Since there are no K_V_4 channel activators, targeting auxiliary subunits is presented as a promising therapeutic alternative. These proteins not only regulate the activity of K_V_4 channels, but also intervene in their assembly and transport to the cell membrane. Interestingly, in this context, the compound NS5806 has been shown to potentiate currents through K_V_4 channels in a ChiP2-dependent manner, in addition to attenuating allodynia in a neuropathic pain model [247,248].

On the other hand, some studies have documented the residual expression of K_V_2.1 after the pain phenotype has been established, which could be therapeutically exploited by activators of these channels to increase their conductance. Indeed, retigabine, a positive allosteric modulator of K_V_2–K_V_5 channels, increases these currents and reduces nerve transmission through Aδ and C fibers to the dorsal horn of the SC [242], in addition to producing the hyperpolarization of primary afferent fibers [249]. As a consequence of these effects, it has been suggested that retigabine could exert an analgesic action in some models of neuropathic pain [238,242,250].

It should be noted that retigabine can also increase M currents besides acting on K_V_2 channels. In this case, the compound causes a shift in the K_V_7 channel conductance–voltage curve in the hyperpolarizing direction, which is associated with an increase in the channel’s opening time and a significant decrease in its deactivation kinetics [251,252,253]. However, due to its side effects, clinical use of retigabine has been discontinued. A therapeutic alternative has emerged with flupirtine, a structural analogue of retigabine. Flupirtine also shares this mechanism of action, acting on K_V_7 channels, although it has also been shown to enhance analgesia mediated by GABA_A_ receptors [254,255,256].

## 5. Voltage-Gated Chloride and Proton Channels

### 5.1. Cl^−^ Channels and Nociception

The excitability of primary afferent neurons has been traditionally associated with cation fluxes across the plasma membrane, key determinants in the generation and propagation of action potentials, as well as in their ability to respond to tissue-damaging stimuli. However, recent research has highlighted the role of chloride (Cl^−^) homeostasis and its fluxes across the cell membrane in nociception. In particular, these processes are gaining relevance due to their involvement in the development and maintenance of neuropathic pain. Recent studies suggest the implication of alterations in chloride channel activity and Cl^−^ homeostasis in primary afferent nociceptors [257].

It is worth mentioning that the intracellular concentration of Cl^−^ ions is regulated by transmembrane transporters, such as the Na^+^-K^+^-2Cl^−^ cotransporter 1 (NKCC1) and K^+^-Cl^−^ cotransporter 2 (KCC2). The deregulation of these transporters under pathological conditions can lead to an increase in intracellular chloride levels, which can facilitate the depolarization of nociceptive neurons and increase pain signaling [257]. The changes caused in chloride homeostasis and the equilibrium potential of the ion by the deregulation of membrane transporters can be so drastic that they can cause the neurotransmitter GABA acting on its extrasynaptic receptors to have a depolarizing and, therefore, pronociceptive effect [258].

Therefore, most studies focus on the role of chloride homeostasis and other chloride channels, such as calcium-activated or ligand-gated channels and GABA_A_ receptors, respectively, and the NKCC1 and KCC2 transporters, in modulating nociceptor excitability and pain perception. However, this exciting topic falls outside the scope of this review, as it is centered on voltage-gated channels.

Likewise, the ClC-2 channel, a specific type of voltage-gated chloride channel, has been associated with nociception due to its altered expression and activity in certain pain states beyond neuropathic pain. This channel belongs to a large family of proteins encoded by nine different genes in mammals. Based on sequence homology, these channels can be grouped into three branches. The first branch comprises five channels expressed in the cell membrane and corresponds to the ClC-0, ClC-1, ClC-2, ClC-Ka, and ClC-Kb channels. In contrast, the proteins encoded by the other two branches reside predominantly in intracellular membranes [259].

ClC-2 channels exhibit a symmetrical homodimeric structure, with each subunit containing an independent pathway for ion permeation. The transmembrane domain generates two chloride-conducting pores with an independent gating mechanism. The activation of ClC-2 channels occurs through electrostatic and steric repulsion when intracellular chloride ions occupy the pore, inducing conformational changes in the residues responsible for gating. Unlike most ClC family homologs, ClC-2 is activated by hyperpolarization rather than depolarization, making it unique in its physiological roles [259]. However, the chloride equilibrium potential may be altered under neuropathic pain conditions [257]. As a result, the activation of these channels could depolarize the cell membrane and increase its excitability, a mechanism similar to that observed with extrasynaptic GABA_A_ receptors [258].

Although the direct role of the ClC-2 channel in neuropathic pain is yet to be determined, it could play a key role in regulating synaptic inhibition by controlling chloride gradients at the spinal level in the nervous system [260,261]. These gradients directly affect GABAergic neurotransmission and, therefore, contribute to the pathophysiology of neuropathic pain.

### 5.2. Proton Channels and Neuropathic Pain

While voltage-gated H⁺ channels (particularly the Hv1 subtype) have been implicated in the pathogenesis of neuropathic pain through microglial regulation and inflammatory signaling, their mechanistic roles remain poorly characterized. Emerging evidence indicates that Hv1 mediates nociceptive signaling by amplifying microglial reactive oxygen species production and potentiating the release of proinflammatory cytokines in the central nervous system.

Voltage-gated H⁺ channels allow for the flow of protons across the cell membrane in response to changes in electrical potential and play important roles in various cellular functions. When activated during membrane depolarization, they allow proton efflux, thus helping to maintain the acid–base balance within cells. Furthermore, Hv1 proton channels may directly influence neuronal excitability by conducting ions that depolarize the neuronal membrane. As mentioned above, they may also indirectly modulate excitability through their effects on ROS production, pH regulation, injury, and microglial activity.

The molecular structure of Hv1 channels differs from other voltage-gated ion channels due to their unique composition and function [262]. These channels consist solely of a voltage-sensing domain (VSD) composed of four transmembrane helices (S1–S4) and an additional amphipathic helix (S0) at the N-terminus. They are composed of two subunits, each with its own proton permeability pathway within the VSD [262,263]. The S4 helix in the VSD is crucial for sensing changes in transmembrane voltage. It undergoes outward displacement in response to depolarization, which changes the internal salt bridge network and reconfigures the proton permeability pathway [262]. The two subunits of Hv1 channels interact during the channel opening process, showing a positive cooperativity that modulates the voltage response of the two permeation pathways [264]. Furthermore, Hv1 channels exhibit unidirectional conductance, allowing protons to exit the cell but not enter it, which is essential for maintaining intracellular pH homeostasis.

The involvement of ROS in the development of neuropathic pain is reinforced by the fact that, following spinal cord injury, a large proportion of patients develop neuropathic pain [265]. Furthermore, NOX2-derived ROS in microglia have been associated with neuropathic pain induced by nerve injury [266]. It is also known that, in response to peripheral nerve injury, macrophages are recruited by DRGs and increase ROS production through a NOX2-dependent mechanism. Interestingly, NOX2^−/−^ mice display a reduced neuropathic pain phenotype [267]. Given the established link between Hv1–NOX and NOX2 and the development of neuropathic pain, it was initially suggested that microglial Hv1 channels could initiate and maintain neuropathic pain after spinal cord injury [268].

Indeed, Hv1 channels are functionally expressed in spinal cord microglia and show significant upregulation following peripheral nerve injury [269]. Furthermore, the activation of these channels contributes to the onset of neuropathic pain by favoring the production of reactive oxygen species (ROS). This production of ROS is associated with astrocyte activation, which worsens pain sensitivity [269]. Likewise, Hv1-null mice display decreased pain sensitivity after nerve injury compared to wild-type mice. Together these data suggest that Hv1 channels are relevant in mediating pain responses through microglial and astrocytic interactions.

## 6. Voltage-Gated Ion Channel Dysregulation in Supraspinal Pathways

Pain transmission involves complex neural circuits that run from the periphery to the brain. While spinal mechanisms are crucial for the initial processing of nociceptive signals, supraspinal pathways, predominantly cortical and brainstem circuits, are essential for the integration, modulation, and conscious perception of pain.

The cortex plays a central role in pain perception, integrating sensory, emotional, and cognitive aspects. Key regions include the somatosensory cortices (S1 and S2), which are responsible for the sensory-discriminative aspects of pain, such as location and intensity. S1 receives nociceptive inputs from the thalamus, while S2 integrates information from multiple body regions. The insula and anterior cingulate cortex (ACC) provide pain’s affective and emotional dimensions. The insula contributes to the subjective experience, while the ACC is related to emotional and motivational responses. The prefrontal cortex participates in cognitive pain evaluation, including attention and modulation. Its dysfunction is associated with chronic pain.

The brainstem bridges cortical centers and spinal circuits, modulating pain through several structures, including the periaqueductal gray matter that integrates descending pain control signals, producing analgesia by inhibiting nociceptive neurons. The rostral ventromedial nucleus of the medulla oblongata contains neurons that facilitate and impede pain, exerting bidirectional control. Finally, the parabrachial area involves pain’s affective and motivational aspects and contributes to the transition from acute to chronic pain.

While alterations in peripheral voltage-gated ion channels predominate in initiating neuropathic pain, supraspinal mechanisms may maintain pain through central sensitization and altered descending control. However, direct evidence for the involvement of voltage-gated ion channels in supraspinal pathways is scarce and contrasts with their well-documented contribution to peripheral and spinal mechanisms. In this context, it is worth noting that neuropathic pain may involve important changes in neuronal excitability and synaptic transmission along the peripheral and supraspinal pathways. Indeed, a key event in the development of neuropathic pain is central sensitization, which involves alterations in neuronal excitability and synaptic transmission. Though the involvement of voltage-gated ion channels has not been directly documented in the supraspinal context, their contribution to neuronal excitability suggests that they may be indirectly involved in neuropathic pain, as discussed below. 

It has been documented that Na_V_1.3 channels, especially in the thalamus, can generate and maintain neuropathic pain due to their role in central hyperexcitability [14,201]. Particularly, after a spinal cord injury, Na_V_1.3 channel expression increases significantly in thalamic neurons, which is associated with increased spontaneous neuronal activity contributing to pain generation. Furthermore, it has been observed that spinal cord injuries can trigger supraspinal changes in Na_V_ channel expression in thalamic neurons [14,37,270].

Specifically, four weeks after injury, immunostaining for Na_V_1.3 channels was significantly increased in neurons of the ventral posterolateral nucleus of the thalamus. Electrophysiological recordings from neurons in this region in SCI animals showed a high rate of spontaneous activity, independent of ascending afferent input. Interestingly, antisense oligonucleotides targeting Na_V_1.3 channel messengers reduced their expression in the thalamus and reversed the increase in spontaneous activity. Furthermore, the exacerbated spontaneous activity persisted even after complete spinal cord transection, indicating that afferent input is not essential for maintaining thalamic hyperexcitability, suggesting that this region may function as an intrinsic pain generator [37,270] and supporting the finding that Na_V_1.3 overexpression in the post-spinal cord injury thalamus contributes to spontaneous neuronal activity and neuropathic pain. Although further research is needed to fully understand Na_V_ channels’ contributions to neuropathic pain, their involvement in central sensitization and hyperexcitability suggests a potential role in the molecular mechanisms of the condition.

On the other hand, T-type calcium channels, especially those of the Ca_V_3.2 class, are not only involved in neuropathic pain at the peripheral level, but also appear to play a role at the supraspinal level. Specifically, it has been found that Ca_V_3.2 channels are expressed in GABAergic neurons and contribute to high-frequency firing activity in the reticular thalamic nucleus and anterior pretectum (APT), a region involved in pain perception [162,271]. This activity is increased in animal models of neuropathic pain, while the specific elimination of Ca_V_3.2 channels in APT neurons reduces mechanical allodynia.

Likewise, the upregulation of T-type channels in the anterior cingulate cortex (ACC) has been shown to alleviate neuropathic pain [272]. After chronic nerve constriction injury, the upregulation of Ca_V_3.2 channels has been observed in the ACC of rats, suggesting that these channels may be involved in the development of neuropathic pain. These findings are associated with a significant increase in T-type calcium currents, which results in increased neuronal activity in the ACC. Furthermore, the T-type channel inhibitor NNC 55-0396 reduced the frequency of postsynaptic excitatory synaptic currents (mEPSCs) and reduced neuronal firing frequency in the ACC [272]. Finally, a microinjection of the T-channel inhibitor into the ACC partially alleviated mechanical and thermal allodynia in rats with neuropathic pain. Taken together, these data suggest that T channels, particularly those of the Ca_V_3.2 class, could contribute to neuronal hyperexcitability in this region and, thus, participate in the development of neuropathic pain.

Finally, like Na_V_ and Ca_V_ channels, K_V_ channels in supraspinal pathways are less well-characterized than their spinal and peripheral counterparts. While further research is needed to clarify their role in specific brain regions, some evidence supports their possible involvement in neuropathic pain through cellular mechanisms such as thalamocortical hyperexcitability and microglial activation.

K_V_7 channels regulate neuronal excitability in the thalamus and the ACC, among other brain regions [273,274]. Furthermore, alterations in the functional expression of these proteins at the supraspinal level are recognized as contributing to central sensitization, a central feature of neuropathic pain. As mentioned earlier, retigabine and flupirtine, K_V_7 channel activators, effectively prevent neuropathic pain in animal models by reducing neuronal excitability [229,255,256]. Thus, by activating K_V_7 channels, these drugs reduce the heightened neuronal activity associated with the condition, suggesting a possible role for these channels in pain processing at the supraspinal level.

Likewise, K_V_1.3 channels in spinal cord microglia are implicated in neuroinflammation linked to neuropathic pain [275]. Studies in animal models show that neuropathic pain is associated with an increased expression of M1 (proinflammatory) and M2 (anti-inflammatory) microglial phenotypes in the spinal cord, along with elevated levels of NLRP3 inflammasome components (NLRP3, caspase-1, and IL-1β), markers of neuroinflammation. The inhibition of K_V_1.3 with PAP-1 reduced hyperalgesia, M1 polarization, and the expression of NLRP3, caspase-1, and IL-1β, suggesting that K_V_1.3 channels may play a key role in neuropathic pain by promoting M1 microglial polarization and activating the NLRP3 inflammasome [275].

In this context, the scorpion-venom-derived peptide αKtx12 has been investigated for its neuroprotective potential by acting as a selective K_V_1.3 channel blocker [276]. Studies show that αKtx12 increases the viability of SH-SY5Y neuroblastoma cells and promotes hippocampal pyramidal cell proliferation in animal models. Furthermore, the peptide reduces microglial activation and the production of proinflammatory cytokines, generating a neuroprotective environment. Although this mechanism is spinal, similar K_V_1.3-mediated activation could occur in supraspinal regions such as the periaqueductal gray, even though direct corroborating evidence is still lacking. These findings suggest that K_V_1.3 inhibition by αKtx12 might be considered an alternative for treating neuropathic pain associated with nervous system injuries.

## 7. Genetic Defects in Voltage-Gated Ion Channels Associated with Neuropathic Pain

Although neuropathic pain is defined as an event caused by nerve disease or injury, a few channelopathies could fit this framework, because they represent a form of nerve dysfunction at the molecular level. Therefore, some mutations in voltage-gated ion channels, specifically Na_V_ channels, which alter the normal electrical properties of nerves, can cause neuronal hyperexcitability and generate pain signals, even without apparent structural nerve damage. Therefore, in these cases, the underlying “lesion” is genetic and functional rather than anatomical.

As mentioned earlier, genetic alterations in Na_V_ channels may cause neuropathic pain. These mutations disrupt channel function, leading to the hyperexcitability of nociceptive neurons and contributing to various painful conditions. For example, gain-of-function mutations exist in *SCN9A* and *SCN10A*, which encode the Na_V_1.7 and Na_V_1.8 channels, respectively [277,278,279,280]. These mutations are associated with inherited erythromelalgia (IEM) and paroxysmal extreme pain disorder (PEPD), both characterized by severe spontaneous pain due to the hyperexcitability of nociceptive neurons [277,279,281,282,283].

Na_V_1.7 mutations associated with IME cause a hyperpolarizing shift in activation and slower channel deactivation. These changes lower the threshold for generating action potentials in sensory neurons, increasing their excitability [279,284,285]. Furthermore, the mutations enhance the response of Na_V_1.7 to slow depolarizations, amplifying small-input signals close to the resting potential. Because Na_V_1.7 is primarily expressed in small sensory and nociceptive neurons, these alterations contribute to the chronic pain experienced by IME patients [279].

Generally speaking, these mutations alter the biophysical properties of Na_V_ channels, resulting in a reduction in the activation threshold, with a consequent increase in neuronal excitability and the spontaneous firing of nociceptive neurons. These changes give rise to characteristic symptoms of neuropathic pain, such as spontaneous pain, hyperalgesia, and allodynia. Understanding these genetic defects provides insight into the molecular basis of neuropathic pain and guides the development of targeted therapies, such as selective Nav channel inhibitors.

On the other hand, genetic defects in Ca_V_ and K_V_ channels linked explicitly to neuropathic pain, without association with different types of pain, are not well defined. Although these channels play a fundamental role in neurotransmitter release and neuronal excitability and alterations in their function are implicated in chronic and neuropathic pain, most evidence points to their involvement in a wide spectrum of painful conditions, and not exclusively in neuropathic pain. Thus, although Ca_V_ and K_V_ channel dysfunction is mechanistically crucial in neuropathic pain, it has not been established that any specific genetic alteration in these channels causes neuropathic pain independently of other pain or neurological conditions.

## 8. Neuropathic Pain Trials: Testing Ion-Channel-Targeting Drugs

The landscape of clinical trials for voltage-gated ion channel antagonists or blockers for the treatment of neuropathic pain, although rapidly expanding, is currently still limited. The clinical trial pipeline is growing for Na_V_ channels, with several pharmaceutical companies developing candidates targeting Na_V_1.7 and Na_V_1.8 subtypes, which are critical in pain transmission. In particular, Na_V_1.8 channel blockers have shown potential, as Na_V_1.8 is primarily expressed in peripheral sensory neurons involved in pain transmission and is less likely to cause side effects in the central nervous system [286]. A partial blockade of Na_V_1.8 has been shown to reduce neuronal hyperexcitability significantly in models of chronic neuropathic pain. As previously mentioned, one notable compound is VX-548, a selective Na_V_1.8 blocker approved by the FDA for moderate to severe acute pain, being studied for its potential in neuropathic pain [73,74]. It offers an alternative to opioids, with a favorable side effect profile and no addictive potential.

On the other hand, Na_V_1.7 channel inhibitors have faced challenges regarding selectivity and efficacy, though research continues on these compounds as promising non-opioid treatments for neuropathic pain. Some trials have shown promising results; however, most candidates have failed in advanced phases due to efficacy issues and study design discrepancies [287]. One such trial specifically focused on vixotrigine (BIIB074) for treating trigeminal neuralgia [286,288]. However, its Phase II trial for neuropathic pain was discontinued [286]. Therefore, many research teams and companies are prioritizing Na_V_1.8 inhibitors, following the stalling of Na_V_1.7 trials.

There are also ongoing clinical trials of Ca_V_ channel blockers or antagonists for treating neuropathic pain, specifically targeting Ca_V_2.2 (N-type) channels. Thus, the novel compound C2230, which potently and selectively blocks these channels [184], has been documented to have analgesic effects in multiple pain models, including neuropathic pain, through systemic and intranasal administration without generating tolerance or adverse behavioral effects. This compound is considered to be a promising candidate for further clinical development [289]. Other Ca_V_2.2 channel blockers, such as RD2, have shown efficacy in animal models of neuropathic pain and a good tolerability in healthy human volunteers, reinforcing the potential for targeting these channels [205,289,290]. In addition to Ca_V_2.2 channels, Ca_V_3 channel blockers, such as ABT-639 [291], have also been studied in clinical trials for diabetic peripheral neuropathic pain. However, more recent reviews and data indicate that ABT-639 and other similar T-type channel blockers have not advanced further in clinical development for neuropathic pain due to their lack of efficacy [292].

Furthermore, K_V_ channel activators, particularly targeting K_V_7 subtypes (KCNQ), are under active preclinical and clinical investigation for neuropathic pain. Novel compounds such as SCR2682 and advances in chemical optimization highlight the potential of this approach to deliver novel non-opioid therapies for chronic neuropathic pain. This compound potentiates M currents in dorsal root ganglion neurons and reduces nerve-injury-induced pain in animal models, showing potential as a therapeutic tool [293,294]. Research also focuses on overcoming toxicity and improving selectivity by modifying compounds like retigabine. The modulation of K_V_ channels, especially K_V_7, remains a promising strategy for treating neuropathic pain, with ongoing efforts to develop safer and more effective activators. Other K_V_7 activators, such as retigabine, approved for epilepsy but limited by side effects, and newer compounds, such as ASP0819, have been explored in preclinical studies and early clinical trials to improve selectivity and reduce toxicity in pain management [201].

Likewise, BHV-7000 is a late-stage drug candidate that selectively activates K_V_7.2/7.3 potassium channels, primarily developed for epilepsy and mood disorders, with ongoing exploration for pain treatment [295]. Its clinical development highlights its potential to regulate neuronal hyperexcitability with improved safety and tolerability profiles. Interestingly, gabapentin, widely used for neuropathic pain due to its binding to the Ca_V_α_2_δ auxiliary subunit of HVA-type Ca_V_ channels, has also been found to activate K_V_7 channels [296], suggesting that part of its analgesic effect may involve K_V_ channel modulation.

## 9. Challenges and Future Directions

### 9.1. Key Hurdles in Ion Channel Research for Neuropathic Pain

There are still significant challenges in investigating voltage-gated ion channels in neuropathic pain. The first of these is related to the complexity of the disease. Neuropathic pain is a multifactorial and heterogeneous condition with diverse etiologies and multiple cellular and molecular mechanisms. Although Na_V_ channels are the most studied, their dysfunction is detected in <20% of patients, suggesting that other ion channels and mechanisms are also involved. This complexity hampers the identification of universal molecular targets and the development of effective therapies.

Second, there is a great diversity of ion channels. As detailed throughout this manuscript, multiple types of voltage-gated ion channels, each with distinct expression patterns and functions, are implicated in the pathophysiology of neuropathic pain. The redundancy and overlap among these channels complicate efforts to identify the most relevant candidates and design selective modulators without off-target effects.

There is also a significant translational gap between animal models and humans. Many of the findings on the involvement of voltage-gated ion channels in neuropathic pain come from animal models, which may not fully replicate the human disease. Differences in ion channel expression and regulation may limit the translation of preclinical findings into effective clinical treatments.

On the other hand, nonselective ion channel blockers can be effective in animal models, but they often have a limited clinical efficacy and cause adverse effects. Therefore, developing more selective compounds that target peripheral channels without central or cardiac impacts remains a significant challenge. Likewise, following nerve injury, the functional expression of voltage-gated ion channels can change dynamically, influenced by inflammatory mediators and neuronal activity. Understanding these temporal and spatial changes is crucial for producing effective therapeutic responses.

Lastly, the proteins that form voltage-gated ion channel complexes are targets of post-translational modifications such as phosphorylation, ubiquitination, SUMOylation, and glycosylation, which affect their activity, intracellular trafficking, and half-life. Studying how these processes are affected during the development of neuropathic pain would be a topic of interest for future studies, especially considering its therapeutic potential (see below). Furthermore, it would be of great interest to expand our knowledge of how the release of cytokines and chemokines during neuroinflammation can affect the function and expression of ion channels and how these processes can contribute to the molecular pathophysiology of neuropathic pain. Likewise, studying how DNA methylation or histone acetylation and other epigenetic modifications can affect the expression patterns of ion channels is another topic of great relevance, since these modifications can affect pain perception and promote the development of neuropathic pain.

### 9.2. Small Molecules Versus Biologics in the Future of Voltage-Gated Ion Channel Therapies in Neuropathic Pain

Small molecules remain the primary and most promising approach to targeting voltage-gated ion channels in neuropathic pain. Recent advances include highly selective inhibitors for Na_V_ and Ca_V_ channels, with successful clinical trials generating significant momentum for this approach. Small molecules offer advantages such as oral bioavailability, ease of synthesis, and the ability to modulate channel activity or trafficking in a reversible and tunable manner.

Innovative small-molecule strategies are emerging, such as targeted protein degradation (PROTACs), which can selectively degrade specific ion channel subtypes and potentially offer longer-lasting effects [206,207,208,297]. Furthermore, small molecules are being developed to disrupt protein–protein interactions critical to channel function and trafficking, expanding the scope of therapeutic mechanisms. PROTACs in the ion channel field represent a novel strategy to modulate ion channel function by targeting the channel proteins rather than through traditional inhibition, potentially offering increased specificity and overcoming drug resistance mechanisms. They are small, heterobifunctional molecules designed to selectively degrade target proteins by exploiting the cell’s ubiquitin–proteasome system (UPS). They are composed of two ligands connected by a linker—one ligand binds to the protein of interest, and the other recruits an E3 ubiquitin ligase. This proximity induces the ubiquitination of the target protein, marking it for degradation by the 26S proteasome, effectively reducing the cellular levels of the protein rather than simply inhibiting its function [206,207,208].

Na_V_1.7 and Na_V_1.8 are also susceptible to PROTAC-mediated targeted protein degradation. Engineered PROTACs have been used to induce the rapid and near-complete degradation of these channels in vitro, revealing that Na_V_ channels can be targeted intracellularly by PROTACs for degradation via the proteasome rather than the lysosomal pathway.

Indeed, developing new therapeutic strategies for neuropathic pain has also found a promising target in the Na_V_1.7 and Na_V_1.8 channels. Recent research has shown the feasibility of selectively degrading these channels using small molecules to induce targeted protein degradation. To achieve this, degron-labeled systems, such as dTAG, fused to Na_V_1.7 and Na_V_1.8 channels have been used, allowing for the evaluation of the efficacy of different PROTACs that recruit E3 ligases to label the protein targeted for degradation [298]. Chimeras have been designed with degron tags at both the C- and N-termini of the channels, observing that the C-terminal location favors more efficient degradation. The recruitment of E3 ligases such as CRBN and VHL effectively mediates degradation, showing vigorous activity for Na_V_1.8. Furthermore, degradation is proteasome-dependent, as it is blocked by specific inhibitors such as MG132, but not by lysosomal inhibitors [298].

These findings represent the first experimental evidence for the small-molecule-directed degradation of ion channels, providing a significant framework for the pharmacology of Na_V_ channels related to neuropathic pain. The therapeutic potential of these findings is considerable, as the precise control of Na_V_ channel abundance in preclinical models using these techniques complements existing genetic tools and will pave the way for the development of novel non-opioid analgesics.

On the other hand, biologics, including peptide toxins, have demonstrated efficacy in preclinical models and some clinical trials, especially for calcium channels. These agents can offer specificity but often face administration route, stability, and production challenges. Although they represent innovative tools and can inspire new drug designs, their widespread adoption is limited compared to small molecules.

Given the complexity of neuropathic pain, future advances could also come from rational combination therapies, using small molecules, biologics, or both to target multiple pathways simultaneously. Specifically, integrating molecular engineering techniques, mechanistic analysis, and pharmacology opens new avenues for the selective modulation of key proteins involved in pain molecular pathophysiology, with a potentially transformative impact on the medical management of neuropathic pain.

## Data Availability

Not applicable.
